# Making Ends Meet or Just Meeting at the Ends? Assessing End-to-End Distance in Folded RNA Sequences and Other Branched Structures

**DOI:** 10.1007/s11538-026-01718-z

**Published:** 2026-08-19

**Authors:** Torin Greenwood, Christine Heitsch

**Affiliations:** 1 Department of Mathematics, North Dakota State University, 1210 Albrecht Boulevard, Fargo, ND 58102 USA; 2 School of Mathematics, Georgia Institute of Technology, 686 Cherry Street, Atlanta, GA 30332 USA

**Keywords:** Dyck, Motzkin, Pfold, Stochastic context-free grammar, Analytic combinatorics, RNA, Secondary structures

## Abstract

Researchers have repeatedly found that the ends of an RNA sequence are significantly closer than expected for a random linear chain. However, we prove that the ends of a branched structure are almost certainly close. Our results are obtained via combinatorial branching models of increasing complexity using tools from multivariate analytic combinatorics. We completely characterize parameters tracking end-to-end distance, including means and variances. Then, we compare to existing datasets of known rna structures, as well as the minimum free-energy structures of randomized shuffles. We find that the shuffled structures resemble our theoretical distributions while the known rna structures have similar parameter values but are more concentrated. The helices on the exterior loop of a structure draw the ends of the sequence together, and we find evidence that these helices are more stable than expected when compared to their counterparts in uniformly random branched structures.

Rna molecules are not merely passive carriers of genetic information but play direct roles in many of the cell’s essential processes. The function of an rna molecule depends critically on the three-dimensional conformation induced by intramolecular base pairing. These interactions generate characteristic structural motifs that govern stability, specificity, and interaction with proteins and other nucleic acids. Identifying the three-dimensional structure can be technically challenging, time-consuming, and often infeasible. Instead, it is more tractable to identify a two-dimensional approximation called the *secondary structure*, which still captures essential functional information. Secondary structures are a special type of branching structure, with several interpretations defined below. In this work, we consider only rna molecules composed of a single strand.

Recent papers in rna folding have concluded that the ends of rna molecules often end up close to one another. Here, we ask: how common is this for a random branched structure? To address this problem with mathematical rigor, we consider three increasingly complex distributions over branched structures and five different parameters measuring *end-proximity*. By using an analysis pipeline from analytic combinatorics, we find complete descriptions of the limiting distributions of each parameter. We find uniformly small means for each parameter, indicating that the endpoints of branched structures are “necessarily” close, rather than this being a feature unique to thermodynamically favorable rna secondary structures (Yoffe et al. 2010).

Our contribution to the increasing body of work on end-proximity has three main advancements: first, we refine the analytic combinatorics pipeline to give limiting probability generating functions (PGFs) describing the distributions of each parameter. Previous work focused on the mean of each parameter, but our analysis allows us to identify variances, which illustrate a key difference between random branched structures and known rna families. Next, we are able to analyze the joint distribution of multiple characteristics of end-proximity simultaneously. Besides being of interest in its own right, the joint distribution unlocks our ability to analyze more refined definitions of end-to-end distance (Aalberts and Nandagopal 2010). Finally, we can study the distribution of branched structures output by the Pfold grammar (Knudsen and Hein 2003). This grammar has been shown to predict rna structures with high accuracy.

**Fig. 1:**
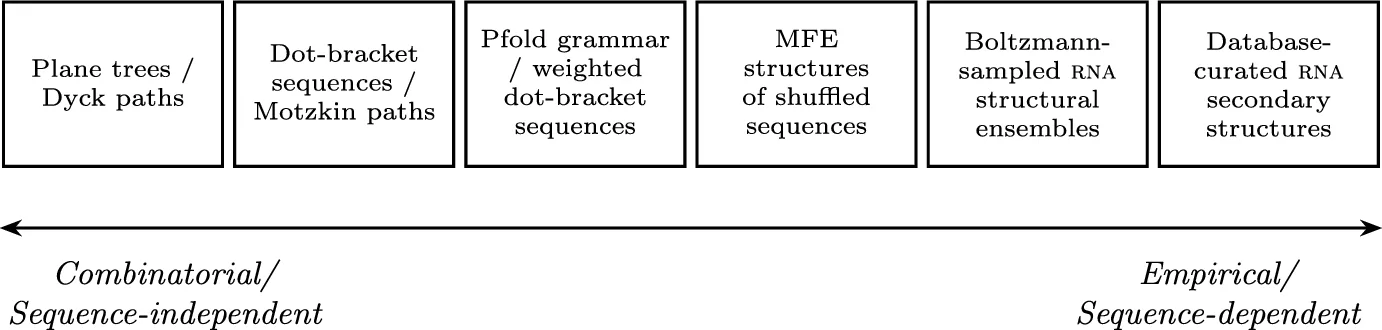
The spectrum of rna secondary structure models we consider. Our three theoretical models are on the left, described in Sect. 1. The main results on the models appear in Sect. 3 and Sect. 7, with visualizations of the measures of end proximity in Sect. 4. Our comparisons to rna secondary structures derived from real rna sequences appear in Sects. 5,  6, and 8

In Sect. 1, we give an initial outline of the three theoretical branching models considered, as depicted on the left in Fig. 1. We state our main theoretical results on the models in Proposition 1. Then, in Sect. 2, we describe the primary tool we develop in analytic combinatorics to extract distributions. Following this is our mathematical analysis of each characteristic of end-proximity in Sect. 3 and a description of the limiting distributions in Sect. 4. We shift to analyzing data derived from real rna sequences and secondary structures in Sects. 5 and 6, matching the three right boxes in Fig. 1. To investigate the pairing propensity of the ends of a sequence, we analyze the lengths of the first helix and first stem of a structure in our theoretical models in Sect. 7. In Sect. 8 we compare these results to our datasets of structures to conclude that the sequences indeed make ends meet. Finally, in Sect. 9, we indicate implications to combinatorics and directions for future work.

## The Distributions of End-Proximity

The one-dimensional structure of an rna molecule, known as its sequence, is an oriented linear polymer composed of four nucleotides: a(denine), c(ytosine), g(uanine), and u(racil). The links of this chain are formed by covalent bonds which connect the 3’-hydroxyl group of one sugar to the 5’-phosphate group of the next nucleotide. This creates a strong 5’–3’ linear polymer with an alternating sugar-phosphate backbone and nitrogenous bases available for pairing.

The intra-sequence base pairing of an rna molecule creates a two-dimensional branched structure, known as its (pseudoknot-free) secondary structure.^1^ Hydrogen bonds create a bridge between complementary nucleotides; the familiar Watson-Crick-Franklin pairings of c g/g c and a u/u a along with the wobble g u/u g one are known as the canonical base pairs.^2^ An rna secondary structure decomposes into runs of consecutive base pairs, known as helices, and single-stranded regions called loops. Our focus here is the *exterior loop* which is the set of nucleotides (paired and unpaired) not contained within any base pair.

Specifically, we consider the “end-to-end” (ETE) distance in rna molecules as the shortest path from the 5’ to 3’ ends. In a secondary structure, this relates to one less than the number of nucleotides in the exterior loop. However, to compare with experimental measurements, such as force microscopy (Gerland et al. 2001) or FRET (Leija-Martínez et al. 2014; Lai et al. 2018), this discrete count must be translated into an estimate of the physical distance. Hence, it is important to distinguish between the covalent links in the sugar-phosphate backbone and the hydrogen bridges created by base pairing as the latter are ∼2.5 times longer than the former.

Our method allows us to explicitly consider the two different kinds of steps, and to fully characterize their individual and joint distributions. In particular, we can compute arbitrary moments and hence not only give the asymptotic means, as others have done, but also their variances and visualizations of the complete distributions.

### Overview of Main Results

**Fig. 2:**
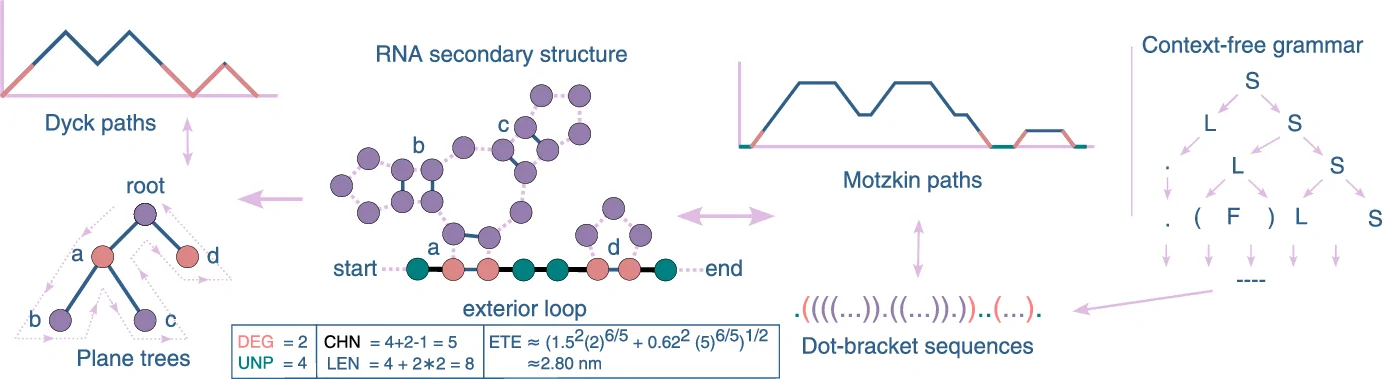
An rna secondary structure, as in the radial diagram (center), consists of runs of stacked base pairs, called helices, separated by single-stranded regions called loops. Since no pairings cross, the arrangement of helices can be abstracted to a plane tree, which is equivalent to a Dyck path under a pre-order walk. A secondary structure is written as a dot-bracket sequence (equivalently a Motzkin path) by reading from left (5’) to right (3’) an unpaired base as a horizontal step (dot), the 5’ side of a base pair as an up step (left parenthesis) and the 3’ side as a down one (right parenthesis). A stochastic context-free grammar, such as the one in Sect. 3.3, generates dot-bracket sequences under a weighting determined by the probability parameters. In this example, there are 8 nucleotides in the exterior loop, with 2 base pairs and 4 unpaired nucleotides which gives an ETE distance estimate of 2.80 nm (Color figure online)

Consider a strand of *n* ordered nodes in a line. An rna secondary structure is a partial pairing of these nodes with arcs above the nodes such that no two arcs cross. Equivalently, rna structures can be represented as dot-bracket sequences (Fig. 2), meaning that they form a *language* $\mathcal {L}$ on the alphabet $\{(, ),.\}$. We consider distributions on $\mathcal {L}$ of varying complexity. When examining increasingly abstract distributions, we find stability in the behavior of end-proximity. This illustrates that end-proximity is a feature of branching structures globally, and not a biological novelty of rna.

As the most rigorous distribution considered, the Pfold grammar (Knudsen and Hein 2003) has been used to predict rna secondary structures with high accuracy. This stochastic context-free grammar (defined in Sect. 3.3) has only three production rules, each with one probability parameter. Nonetheless, it captures biologically meaningful information, such as the stacking of base pairs and the spacing of unpaired nucleotides, in a more mathematically tractable way than the standard thermodynamic recursions used in most secondary structure prediction methods. These recursive production rules assign varying probabilities to each dot-bracket sequence in $\mathcal {L}$.

We obtain a simpler distribution if we assign the same probability to each dot-bracket sequence (or Motzkin path), yielding the uniform distribution over $\mathcal {L}$. One further relaxation is if we condense runs of paired bases into single bracket pairs and omit unpaired bases, which leads to the uniform distribution on balanced parenthesizations, equivalent to Dyck paths. While both distributions have been used elsewhere to analyze rna, the ubiquity of Catalan objects like Dyck paths implies that any characteristics of end-proximity from this model are not due to biological specificities.

We consider four different end-proximity characteristics, summarized in Table 1, of an exterior loop. The number of base pairs is the degree, DEG, equivalently the root degree of a plane tree. The degree DEG is also the number of mountains in a Dyck or Motzkin path, where a mountain is a minimal segment of a path that starts on the horizon, leaves the horizon, and ends back on the horizon. In Motzkin paths, the number of unpaired bases UNP is the number of horizontal steps along the horizon of the path. The number of covalent bonds CHN along the 5’ to 3’ shortest path is related as $\texttt {CHN}= \texttt {DEG}+ \texttt {UNP}- 1$ whereas the total number of nucleotides, denoted LEN, is $2\texttt {DEG}+ \texttt {UNP}$. Hence $\texttt {LEN}- 1 = \texttt {DEG}+ \texttt {CHN}$.

We also consider an approximation of the physical end-to-end distance ETE based on a two-scale self-avoiding freely jointed chain estimate (Aalberts and Nandagopal 2010; Lai et al. 2018) from polymer theory given by

1 $$\begin{aligned} (b^2 \cdot \texttt {DEG}^{6/5} + c^2 \cdot \texttt {CHN}^{6/5})^{1/2} \end{aligned}$$

with a distance of b=1.5 nm for hydrogen bridges and c=0.62 nm for covalent links. We note that this is strictly larger than the root-mean-square estimate of $a \sqrt{\texttt {LEN}- 1}$ originally considered (Yoffe et al. 2010) based on the path length with an average step size of a=0.75 nm (provided that the structure has at least one base pair).

**Table 1:** Parameters characterizing end-proximity of the exterior loop

Parameter	Definition
DEG	Degree of the exterior loop; number of hydrogen bridges
UNP	Number of unpaired bases in exterior loop
CHN	Number of covalent bonds in exterior loop; $\texttt {DEG}+ \texttt {UNP}- 1$
LEN	Number of nucleotides in exterior loop; $2\texttt {DEG}+ \texttt {UNP}$
ETE	Approximate end-to-end distance in nanometers; $\sqrt{1.5^2 \cdot \texttt {DEG}^{6/5} + 0.62^2 \cdot \texttt {CHN}^{6/5}}$

#### Proposition 1

As the length of a dot-bracket sequence approaches infinity, the limiting distributions of end-proximity are as described in Table 2.

**Table 2:** Limiting distributions for the five end-proximity characteristics from Table 1 under the different secondary structure models considered

Characteristic	Distribution or PGF	Mean	Variance
DEG Dyck paths	$1 + {{\,\textrm{NB}\,}}(2, 1/2)$	3	4
DEG Motzkin paths	$1 + {{\,\textrm{NB}\,}}(2, 1/2)$	3	4
DEG Pfold output	$1 + {{\,\textrm{NB}\,}}(2, (1 + \delta )^{-1})$	1+2δ	$2\delta (1 + \delta )$
DEG Pfold default δ	$1 + {{\,\textrm{NB}\,}}(2, 0.56)$	2.55	2.76
UNP Motzkin paths	${{\,\textrm{NB}\,}}(2, 1/2)$	2	4
UNP Pfold output	${{\,\textrm{NB}\,}}(2, \tfrac{1 - \delta }{1 + \delta ^2})$	$2\tfrac{\delta (1 + \delta )}{1 - \delta }$	$\tfrac{2\delta (1+\delta )(1+\delta ^2)}{(1-\delta )^2}$
UNP Pfold default δ	${{\,\textrm{NB}\,}}(2, 0.14)$	12.39	89.19
CHN Motzkin paths	${{\,\textrm{NB}\,}}(2, 1/3)$	4	12
CHN Pfold output	${{\,\textrm{NB}\,}}\!\left( 2, \tfrac{1-\delta }{1 + \delta }\right)$	$\tfrac{4\delta }{1 - \delta }$	$\tfrac{4\delta (1 + \delta )}{(1 - \delta )^2}$
CHN Pfold default δ	${{\,\textrm{NB}\,}}\!\left( 2, 0.13\right)$	13.95	111.21
LEN Motzkin paths	$\left( \tfrac{u}{u^2 + u - 3}\right) ^2$	8	28
LEN Pfold output	$\left( \tfrac{(1-\delta )u}{(1 + \delta ) - \delta (1 + \delta )u - \delta (1-\delta )u^2}\right) ^2$	$2 + 2\delta + \tfrac{4\delta }{1-\delta }$	$\tfrac{2\delta (\delta ^3 - 3\delta ^2 + \delta + 5)}{(1-\delta )^2}$
LEN Pfold default δ	$\left( \tfrac{0.05u^2}{1.78 - 1.38u - 0.17u^2}\right)$	17.50	138.76
ETE Dyck paths	See Corollary 4	2.89	1.42
ETE Motzkin paths	See Corollary 6	3.08	1.56
ETE Pfold default δ	See Proposition 7	3.83	2.23
HEL Motzkin paths	$1 + {{\,\textrm{NB}\,}}(1, 8/9)$	9/8	9/64
HEL Pfold output	$1 + {{\,\textrm{NB}\,}}(1, 1 - \rho ^2p_3)$	$\tfrac{1}{1 - \rho ^2 p_3}$	$\tfrac{\rho ^2 p_3}{(1 - \rho ^2 p_3)^2}$
HEL Pfold default δ	$1 + {{\,\textrm{NB}\,}}(1, 0.21)$	4.72	17.52

The Pfold distribution depends on δ which is determined by the grammar probability parameters; see Proposition 7. The default δ value is 0.78, and all default parameters are described at the end of the proof of Proposition 7. The first helix length, HEL, is defined and studied in Sect. 7

The first observation is the recurrence of a negative binomial distribution ${{\,\textrm{NB}\,}}(r, p)$. This is often described in terms of coin flipping, where the probability of one head is *p* and ${{\,\textrm{NB}\,}}(r, p)$ models the number of tails (i.e. failures) obtained before *r* heads. Its probability generating function (PGF) is $h(u):= [p/(1 - (1-p)u)]^r$ which has mean r(1-p)/p with variance $r(1-p)/p^2$. The coefficient of $u^k$ in the Taylor series expansion (denoted $[u^k] h(u)$) is the probability that *k* tails were flipped before obtaining *r* heads. With r=2 and a fair coin, the discrete distribution begins as (0.25,0.25,0.1875,0.125,0.078125,…). In contrast to a Gaussian distribution, 89% is within one standard deviation of the mean. Hence, we expect a random Dyck path to have degree 1 or 2 half of the time.

When considering the degree of the exterior loop, the distribution in Dyck and Motzkin paths is the same. This can be explained by projecting Motzkin paths down to Dyck paths by removing all horizontal steps, as described in Appendix A. Another instance of this phenomenon appears in Proposition 9 below.

The degree under Pfold is distributed in the same way as the Dyck and Motzkin paths, except that the probability of a success now depends on δ. Under the default Pfold parameters the negative binomial parameter increases slightly from one half to about 0.56, shifting more probability to lower degrees. For instance, the probability that $\texttt {DEG}= 1$ or 2 increases from 0.5 under the uniform distribution to 0.5896 with subsequent degrees being lower. This is reflected in both a lower mean and lower variance, and also shifts $1 \le \texttt {DEG}\le 5$ from 0.8906 to 0.9373.

In contrast, the unpaired base distributions shift much more between the Motzkin paths and the Pfold grammar. The probability of a ‘failure’ (i.e. adding an unpaired base) increases substantially under the Pfold grammar, whereas it is the same for paired and unpaired bases in the uniform Motzkin weighting. As a consequence, this shifts the balance from paired to unpaired in determining the LEN distribution.

Comparing the distributions between our models reveals that ETE is primarily determined by DEG, with UNP playing a secondary role (see Sect. 4). This is apparent in the small difference between the Dyck and Motzkin distributions for ETE, despite the lack of unpaired bases in the Dyck path model. Moreover, although the Pfold LEN average is more than twice that of the Motzkin paths, with a substantially higher variance, the difference in ETE means is <1 nm as is the variance. Consequently, under Pfold, the most biologically realistic model considered, the theory predicts that a random sequence which folds into a branched structure via paired positions would typically have an ETE distance of about 4 nm with a standard deviation of 1.5 nm.

### Comparison with Related Modeling Results

Our primary goal is to provide a theoretical context for recent experimental results on messenger rna (mrna) and long noncoding rna (lncrna) molecules (Lai et al. 2018). They found that average ETE distance, as measured by Förster resonance energy transfer (FRET) experiments, was always in the range of 5–7 nm for the 10 sequences considered. As we will discuss later, this is not inconsistent with our models, and well below the expectation for unpaired polymers.

The fact that experimental results consistently indicate that the ends of long rna molecules are “close” always attracts attention. It is viewed as “counterintuitive,” “surprising,” and even “rather remarkable” (Ermolenko and Mathews 2020) when compared to the vanishingly small probability that the ends of an arbitrary linear chain are near each other (Yoffe et al. 2010). However, when the pairing of the chain is taken into account, the expected distance drops dramatically.

The first paper to explicitly consider the ETE question in this form Yoffe et al. (2010) gave a theoretical argument for the end-proximity of a randomly self-paired polymer. Their formulation depended on the percentage of paired nucleotides and the average helix length, which were estimated from computational minimum free energy (MFE) predictions to be $\sim 60\%$ and ∼4 respectively. They also considered $\texttt {LEN}- 1$, and calculated that it should be ∼12. Under a root-mean-square (RMS) ETE distance estimate of $a \sqrt{\texttt {LEN}- 1}$ with an estimated average step size of a=3/4 nm, they concluded that the expected ETE was 3 nm. This is almost exactly what our simpler Dyck and Motzkin path models predict under the two-scale distance estimate, and what our Pfold model predicts under theirs.

Subsequently (Clote et al. 2011), the $\texttt {LEN}- 1$ problem was considered based on the standard recursive formulation for rna secondary structures. Using generating functions and analytic combinatorics, as is done here, they concluded that the mean should be lower, i.e. about 6 (depending on parameters; see below). Using the largest mean exterior loop length reported in the average step distance estimate yields an ETE of 2.11 nm. Additionally (Poznanovic and Heitsch 2014), the limiting means of DEG and $\texttt {LEN}- 1$ have also been calculated under the Pfold grammar using analytic combinatorics.

Although not formulated in this way, prior work (Gerland et al. 2001) had recursively computed the size of the exterior loop to model force microscopy experiments. They give an explicit physical distance estimate, but as a function of the forces pulling on a specific rna sequence, so it is not comparable with our results. Others (Hofacker et al. 1998), as part of a comprehensive theoretical analysis of motifs in rna secondary structures, used generating functions to estimate the mean number of base pairs and also of unpaired bases in the exterior loop. Again, using the largest means reported, the two-scale distance estimate yields an ETE of 2.71 nm.

Parameters are an important factor in these analyses. A common one in theoretical models is the minimum hairpin length. This is negatively correlated with LEN, although the differences are small (Clote et al. 2011), i.e. about a nucleotide. In the models we consider, Pfold has a minimum of length 1 but the Motzkin paths could be 0. Another factor is that only some nucleotides can pair in a real rna sequence. Other analyses (Hofacker et al. 1998; Clote et al. 2011) have considered a “stickiness” factor which models the pairing propensity, which again is negatively correlated with $\texttt {LEN}= 2 \texttt {DEG}+ \texttt {UNP}$ and again the differences are small.

Finally, we note that others have also analyzed LEN theoretically (Han and Reidys 2012) using the methodology of generating functions and analytic combinatorics, or shown how to efficiently compute it for a given sequence (Mori et al. 2014) under the NNTM recursions. Additionally, if crossed pairings/pseudoknots are included, the calculation changes considerably (Fang 2011). In particular, the growth rate of LEN with sequence length is no longer negligible. As we will discuss, the effect of pseudoknots in the exterior loop (as seen in small subunit ribosomal rna in Sect. 6) may well be to extend the ETE distance beyond the expected small amount.

## Extracting Asymptotic Distributions

Here, we lay out our main tool from analytic combinatorics that allows us to extract limiting distributions of parameters from generating functions. Our result allows us to track the multivariate distribution of several parameters simultaneously. We view *z* as the variable associated to the size of our objects, and $\textbf{u}$ as the array of variables associated to the parameters.

We define $\textbf{u} = (u_1, u_2, \ldots , u_d)$ and $\textbf{u}^\textbf{k} = u_1^{k_1} \cdots u_d^{k_d}$. We also define $[z^n \textbf{u}^{\textbf{k}}]F(z, u)$ to be the coefficient of $z^n \textbf{u}^{\textbf{k}}$ in the series expansion of *F*(*z*, *u*). Additionally, we let $\textbf{0}$ and $\textbf{1}$ be the vectors of all zeroes and all ones, respectively.

In the context of rna, *z* will track the length of an rna sequence, while $\textbf{u}$ will track the degree of the exterior loop and number of unpaired bases in the exterior loop. For example, if $F(z, \textbf{u})$ enumerates rna secondary structures under some model, then $[z^{100} u_1^{1} u_2^{6}] F(z, \textbf{u})$ would correspond to the number of rna secondary structures of length 100 with an exterior loop of degree 1 containing 6 unpaired bases.

### Proposition 2

Let $F(z, \textbf{u})$ be a multivariate GF where $[z^n\textbf{u}^\textbf{k}]F(z, \textbf{u})$ counts the number of objects of size *n* with parameters equal to $\textbf{k} = (k_1, k_2, \ldots , k_d)$. Suppose $F(z, \textbf{u})$ is of the form

$$ F(z, \textbf{u}) = \frac{-b(z, \textbf{u}) \pm g(z, \textbf{u}) \sqrt{c(z)}}{2a(z, \textbf{u})} $$

with everywhere analytic functions *a*, *b*, *c*,  and *g*. Define $p_{n, \textbf{k}}$ to be the probability that an object of size *n* has parameters equal to $\textbf{k}$ as below:

$$ p_{n, \textbf{k}} = \frac{[z^n\textbf{u}^\textbf{k}] F(z, \textbf{u})}{[z^n]F(z, \textbf{1})}. $$

Additionally, suppose there exists a ρ>0 such that c(ρ)=0 and c(z)≠0 for any other value of *z* with $|z| \le \rho$. Assume that $c'(\rho ), g(\rho , \textbf{1}), a(\rho , \textbf{1}) \ne 0$, and that for all $\textbf{u}$ in a neighborhood of zero: $g(\rho , \textbf{u}) \ne 0$ whenever $\textbf{u}$ has all nonzero coordinates; $a(\rho , \textbf{u}) \ne 0$; and there are no non-removable singularities of $F(z, \textbf{u})$ with $|z| \le \rho$ besides z=ρ. Also, assume $F(z, \textbf{1})$ has no non-removable singularities with $|z| \le \rho$ besides z=ρ. Then,

$$ \lim _{n \rightarrow \infty } p_{n, \textbf{k}} = [\textbf{u}^\textbf{k}] \left( \frac{g(\rho , \textbf{u})}{g(\rho , \textbf{1})} \cdot \frac{a(\rho , \textbf{1})}{a(\rho , \textbf{u})}\right) . $$

### Proof

For any fixed $\textbf{u}$ with nonzero coordinates and $|\textbf{u}|$ sufficiently small, we examine the singular expansion of $F(z, \textbf{u})$ near its closest non-removable singularity to the origin, which by assumption is z=ρ. We can expand each part of *F* separately, obtaining as $z \rightarrow \rho$:

$$ -\frac{b(z, \textbf{u})}{2a(z, \textbf{u})} = -\frac{b(\rho , \textbf{u})}{2a(\rho , \textbf{u})} + O\left( 1 - \frac{z}{\rho }\right) $$

and

$$ \frac{g(z, \textbf{u})\sqrt{c(z)}}{2a(z, \textbf{u})} = \frac{g(\rho , \textbf{u}) \sqrt{-\rho c'(\rho )}}{2a(\rho , \textbf{u})} \sqrt{1 - z/\rho } + O(1 - z/\rho )^{3/2}. $$

Overall, for fixed $\textbf{u}$ sufficiently small with nonzero coordinates and as $z \rightarrow \rho$,

$$ F(z, \textbf{u}) = -\frac{b(\rho , \textbf{u})}{2a(\rho , \textbf{u})} \pm \frac{g(\rho , \textbf{u})\sqrt{-\rho c'(\rho )}}{2a(\rho , \textbf{u})}\sqrt{1 - z/\rho } + O(1 - z/\rho ). $$

By standard transfer theorems of analytic combinatorics (c.f. (Flajolet and Sedgewick 2009, Corollary VI.1)),

$$ [z^n] F(z, \textbf{u}) \sim \frac{g(\rho , \textbf{u})\sqrt{-\rho c'(\rho )}}{2a(\rho , \textbf{u})} \cdot \frac{\rho ^{-n}n^{-3/2}}{\Gamma (-1/2)} $$

and

$$ [z^n] F(z, \textbf{1}) \sim \frac{g(\rho , \textbf{1}) \sqrt{-\rho c'(\rho )}}{2a(\rho , \textbf{1})} \cdot \frac{\rho ^{-n}n^{-3/2}}{\Gamma (-1/2)}. $$

Dividing the two gives that pointwise for each $\textbf{u}$ sufficiently small with nonzero coordinates,

2 $$\begin{aligned} \lim _{n \rightarrow \infty } \frac{[z^n] F(z, \textbf{u})}{[z^n] F(z, \textbf{1})} = \frac{g(\rho , \textbf{u})}{g(\rho , \textbf{1})} \cdot \frac{a(\rho , \textbf{1})}{a(\rho , \textbf{u})}. \end{aligned}$$

Because this holds for all $\textbf{u}$ with nonzero coordinates in a neighborhood of the origin, a multivariate analogue of the Continuity Theorem (Flajolet and Sedgewick 2009, Theorem IX.1) completes the proof. Explicitly, because the functions $p_n(\textbf{u}) = [z^n]F(z,\textbf{u})/[z^n]F(z,\textbf{1})$ are probability generating functions, they satisfy $|p_n(\textbf{u})| \le 1$ for all *u* sufficiently small and all *n*. Then, $p_{n, \textbf{k}}$ can be expressed via the Cauchy Integral Formula, and the Bounded Convergence Theorem allows pushing the limit from Eq. (2) inside the integral to complete the proof. □

## End-Proximity in the Exterior Loop

We now apply Proposition 2 to each of our branching models in order of increasing complexity. Our calculations also appear in a supplementary SageMath worksheet available here:

https://github.com/gtDMMB/EndProximity

All reported numbers are computed to high precision in this worksheet.

### End-Proximity in Plane Trees/Dyck Paths

Dyck paths serve as our most abstract model on $\mathcal {L}$ where the only characteristic of end-proximity is the degree, DEG. The following lemma is a recapitulation of the result in Flajolet and Sedgewick (2009, Example IX.8), but provides a template for using Proposition 2 that we will mimic later.

#### Lemma 3

Consider the uniform distribution over all Dyck paths as $n \rightarrow \infty$. $\texttt {DEG}$ tends in distribution to $1 + {{\,\textrm{NB}\,}}(2, 1/2)$, which has mean 3 and variance 4.

#### Proof

Let *D*(*z*, *u*) be the GF where $[z^n u^k] D(z, u)$ is the number of Dyck paths of length 2*n* with *k* mountains. Then, by the standard recurrence for Dyck paths (see Fig. 8),

$$ D(z, u) = 1 + z (u \cdot D(z, 1)) \cdot D(z, u), $$

where $D(z, 1) = 1 + zD^2(z,1)$ is the standard univariate GF. Solving for *D*(*z*, *u*) yields

$$ D(z, u) = \frac{1}{1 - zuD(z, 1)} = \frac{2}{2 - u + u\sqrt{1 - 4z}} = \frac{2((u - 2) - (u\sqrt{1 - 4z}))}{(2 - u)^2 - u^2(1-4z)}, $$

which meets the conditions of Proposition 2 for *u* sufficiently small and |z|≤1/4. Since the discriminant depends only on *z*, its contribution to 2*a*(*z*, *u*) disappears at the singularity z=1/4. Thus, the distribution of mountains approaches the one encoded by

$$ u \cdot \frac{a(1/4, 1)}{a(1/4, u)} = \frac{u}{(2-u)^2}. $$

This corresponds to the sum of two independent geometric random variables, each with parameter 1/2, shifted by one position. □

The limiting distribution provides complete asymptotic information about the probability of a Dyck path having $\texttt {DEG}= k$ for any *k*. With this, we can compute the moments of $\texttt {ETE}$ to arbitrary precision. For any Dyck path, we interpret $\texttt {UNP}= 0$.

#### Corollary 4

For the uniform distribution over all Dyck paths, the limiting distribution of ETE has mean approximately 2.89 and variance approximately 1.42.

#### Proof

We can find the probabilities in the limiting distribution of DEG by expanding the GF in Lemma 3. To expand, note that the function $1/(1-x)^2$ is the derivative of 1/(1-x), and hence the series for $1/(1-x)^2$ is the derivative of a geometric series. We obtain:

$$ \frac{u}{(2-u)^2} = \frac{1}{2} \frac{\frac{u}{2}}{\left( 1 - \frac{u}{2}\right) ^2} = \sum _{n = 0}^\infty \frac{n}{2^{n + 1}} u^n. $$

By the definition of a PGF, this implies that in the limiting distribution,

$$ \mathbb {P}(\texttt {DEG}= n) = \frac{n}{2^{n + 1}}. $$

Note that this could also have been derived by using the definition of the negative binomial, but these sorts of expansions are useful for the more general bivariate distributions below.

Since we interpret $\texttt {UNP}= 0$ for Dyck paths, our distance formula becomes

$$ \texttt {ETE}= \sqrt{(1.5)^2 \texttt {DEG}^{6/5} + (0.62)^2 (\texttt {DEG}- 1)^{6/5}}. $$

Thus,

$$ \mathbb {E}(\texttt {ETE}) = \sum _{n = 0}^\infty \frac{n}{2^{n + 1}}\sqrt{(1.5)^2 n^{6/5} + (0.62)^2 (n - 1)^{6/5}}. $$

To compute this sum to within 0.001, we must bound the tails of the sum. Note:

$$\begin{aligned} &  T_k:= \sum _{n = k}^\infty \frac{n}{2^{n + 1}}\sqrt{(1.5)^2 n^{6/5} + (0.62)^2 (n - 1)^{6/5}} \le \sum _{n = k}^\infty \frac{n}{2^{n + 1}} \cdot 1.63 n^{3/5}\\ &  \quad \le \frac{1.63}{2}\sum _{n = k}^\infty \frac{n^2}{2^n}, \end{aligned}$$

where we bounded the term under the square root by replacing n-1 with *n* and combining the two terms.

It turns out that the latter sum may be computed explicitly. To do so, we first need some well-known formulas for geometric series and their derivatives when |x|<1:

3 $$\begin{aligned} \frac{1}{1 - x} = \sum _{n = 0}^\infty x^n, \ \ \frac{x}{(1-x)^2} = \sum _{n = 0}^\infty nx^n, \ \ \frac{x(1 + x)}{(1-x)^3} = \sum _{n = 0}^\infty n^2 x^n. \end{aligned}$$

Replacing *x* with 1/2 yields for k≥6:

$$\begin{aligned} \sum _{n = k}^\infty \frac{n^2}{2^n}&= \frac{1}{2^k}\sum _{n = 0}^\infty \frac{(n + k)^2}{2^n} = \frac{1}{2^k} \left( \sum _{n = 0}^\infty \frac{n^2}{2^n} + 2k \sum _{n = 0}^\infty \frac{n}{2^n} + k^2 \sum _{n = 0}^\infty \frac{1}{2^n}\right) \\&= \frac{1}{2^k} \left( 6 + 4k + 2k^2 \right) \le \frac{3k^2}{2^{k}}, \end{aligned}$$

where the k≥6 condition was used only in the last inequality. Hence, $T_k \le 1.63/2 \cdot 3k^2/2^{k}$, and the right side is a decreasing function for k≥3. That means that for all k≥20, $T_k \le 0.001$. Hence, it suffices to approximate $\mathbb {E}(\texttt {ETE})$ to within two decimal places by computing

$$ \sum _{n = 0}^{19} \frac{n}{2^{n + 1}}\sqrt{(1.5)^2 n^{6/5} + (0.62)^2 (n - 1)^{6/5}}, $$

the value of which is 2.893. Similarly, when computing $\mathbb {E}(\texttt {ETE})^2$, the tail is now

$$ T_k = \sum _{n = k}^\infty \frac{n}{2^{n + 1}} \left( (1.5)^2 n^{6/5} + (0.62)^2 (n - 1)^{6/5}\right) \le \frac{2.64}{2} \sum _{n = k}^\infty \frac{n^3}{2^n}. $$

Using the same steps with Eq. (3) and the additional relation $x(1 + 4x + x^2)/(1-x)^4 = \sum _{n = 0}^\infty n^3x^n$, we obtain for k≥9:

$$ T_k \le \frac{2.64}{2} \cdot \frac{1}{2^k} \cdot \left( 2k^3 + 6k^2 + 18k + 26 \right) \le \frac{2.64}{2} \cdot \frac{3k^3}{2^{k}}, $$

where the rightmost function is decreasing for k≥5. Hence, we find that the tail is smaller than 0.001 for k≥27, meaning we can estimate $\mathbb {E}(\texttt {ETE})^2$ to two decimal places with the sum,

$$ \sum _{n = 0}^{26} \frac{n}{2^{n + 1}} \left( (1.5)^2 n^{6/5} + (0.62)^2 (n - 1)^{6/5}\right) , $$

and we obtain $\mathbb {E}(\texttt {ETE})^2 \approx 9.795$. Then, the variance can be computed with the shortcut formula Var$(\texttt {ETE}) = \mathbb {E}(\texttt {ETE})^2 - (\mathbb {E}(\texttt {ETE}))^2 \approx 1.42$, where we note that the precision of $\mathbb {E}(\texttt {ETE})$ needs to be increased in order to guarantee that $(\mathbb {E}(\texttt {ETE}))^2$ has error at most 0.001. □

### End-Proximity in Dot-Bracket Sequences/Motzkin Paths

Dot-bracket sequences provide a more nuanced model of branching. The distributional results for the sum UNP + DEG appeared with a different proof method in Banderier and Wallner (2018, Theorem 3.1).

#### Proposition 5

Consider the uniform distribution over all Motzkin paths of length *n* as $n \rightarrow \infty$. The limiting PGF encoding UNP (*u*) and DEG (*v*) as the length of the sequence tends towards infinity is given by

$$ \frac{v}{(u + v - 3)^2}, $$

corresponding to a joint negative binomial distribution.

#### Proof

Let *M*(*z*, *u*, *v*) be the GF tracking Motzkin paths according to length (*z*), UNP (*u*), and DEG (*v*). The univariate GF *M*(*z*, 1, 1) can be specified using the symbolic method as follows:

$$\begin{aligned} \mathcal {M} = \text{ SEQ }\bigg (\rightarrow \text{ or } \nearrow \mathcal {M} \searrow \bigg ). \end{aligned}$$

In words, this states that any Motzkin path can be decomposed uniquely into a sequence of the following: a horizontal step; or, a single step up followed by a Motzkin path followed by a single step down. Additionally, it is simple to track both DEG and UNP in this specification: each horizontal step corresponds to an unpaired nucleotide, and each occurrence of $\nearrow \mathcal {M} \searrow$ adds one to DEG. In total,

4 $$\begin{aligned} M(z, u, v) = \frac{1}{1 - zu - z^2v M(z, 1, 1)}. \end{aligned}$$

Critically, the discriminant of the univariate GF still determines the contributing singularity of the generating function and hence the distribution. After rationalizing (4), we obtain:

$$ M(z, u, v) = 2\frac{2 - 2uv - v + vz - v\sqrt{(1-3z)(1+z)}}{(2-2uz-v+vz)^2 + v^2(1-3z)(1+z)}. $$

This GF satisfies the conditions of Proposition 2: first, g(z,u,v)=v, and all functions are analytic. Consider the zeroes of the denominator. For |z|≤1/3 and *u* and *v* sufficiently small, (2-2uz-v+vz) is bounded away from zero, implying that the denominator overall is bounded away from zero for |z|≤1/3 and *u* and *v* sufficiently small. Hence, the algebraic singularity z=1/3 is indeed the minimal singularity of the GF in all cases.

Substituting z=1/3 into the denominator causes the (1-3z) term to disappear, leaving us with $a(1/3, u, v) = 4/9(u + v - 3)^2$ in the terms of Proposition 2. Then, substituting into the formula of Proposition 2 completes the proof. □

#### Corollary 6

For the uniform distribution over Motzkin paths, the limiting distributions of DEG, UNP, CHN, LEN, and ETE are as in Table 2.

#### Proof

With the limiting bivariate GF from Lemma 5, the distributions for DEG, UNP, CHN, and LEN are easily recovered. Name the limiting PGF $P(u, v) = v/(u + v - 3)^2$. For DEG, note that substituting 1 into *u* eliminates tracking UNP by combining all terms with the same value of DEG but different values of UNP in the bivariate sum. Hence, DEG has limiting PGF $P(1, v) = v/(v-2)^2$. The negative binomial distribution ${{\,\textrm{NB}\,}}(2, 1/2)$ has PGF $1/(v-2)^2$, so the additional *v* in the numerator shifts the distribution up by one. Similarly, UNP has limiting PGF $P(u, 1) = 1/(u-2)^2$, corresponding exactly to ${{\,\textrm{NB}\,}}(2, 1/2)$.

For $\texttt {CHN}= \texttt {DEG}+ \texttt {UNP}- 1$, we first look for the PGF for $\texttt {DEG}+ \texttt {UNP}$, obtained by treating DEG and UNP the same in the bivariate GF. Equivalently, we make the substitution u=v, which means that the coefficient $[u^k]P(u, u)$ is the sum of all coefficients $[u^m v^n] P(u, v)$ where m+n=k. This yields $P(u, u) = u/(2u-3)^2$, the PGF for $1 + {{\,\textrm{NB}\,}}(2, 1/3)$. We subtract one from this distribution to find CHN.

Next, for LEN, to account for the fact that the degree adds twice as much as any unpaired base, we make the substitution $$v = u^2$$ to obtain $$L(u):= P(u, u^2)$$. This yields the limiting PGF in Table 2. To find the mean, compute $L^{\prime }\left(1\right)$, and to find the variance, compute $$L''(1) + L'(1) - (L'(1)^2).$$

No substitution will give the limiting PGF for ETE, but we can still compute statistics on this distribution to arbitrary precision by first identifying the joint distribution of UNP and DEG. This is a generalization of the proof of the mean and variance of ETE for the Dyck path case, and we give details in the appendix, Appendix B. □

### End-Proximity in the Pfold Stochastic Context-Free Grammar

Successfully used to predict rna secondary structures, stochastic context-free grammars assign a distribution to dot-bracket sequences $\mathcal {L}$. Grammars are defined via a set of recursive rules that begin with a specified start symbol, *S*, and evolve until only the terminal symbols of parentheses and dots remain. The Pfold grammar (Knudsen and Hein 2003) is a particularly successful grammar that uses relatively few rules to achieve a high prediction accuracy (Dowell and Eddy 2004). This grammar is defined with the following rules, where each rule is chosen with a corresponding probability $p_i$ or $q_i$ with $p_i + q_i = 1$:

$$\begin{aligned} &  [1]\ S \rightarrow LS\ (p_1)\ \big |\ L\ (q_1) \hspace{.4 in}[2]\ L \rightarrow (F) \ (p_2)\ \big | \ . \ (q_2) \hspace{.4 in} \\ &  \quad [3]\ F \rightarrow (F)\ (p_3) \ \big |\ LS\ (q_3) \end{aligned}$$

Here, the parameters $p_i$ and $q_i$ can be chosen to fit different families of rna, but default values are given in the original paper (Knudsen and Hein 2003).

In Poznanovic and Heitsch (2014), the authors found that in the output of the Pfold grammar, many common structural features of rna are normally distributed. Curiously, the distributions of some features were connected in ways that are independent of the parameters of the grammar. In contrast, we identify that even in the Pfold grammar, the characteristics of end-proximity have limiting distributions that are again negative binomials. This is again due to a factorization of the discriminant of the corresponding GF. As in the Dyck and Motzkin analyses above, the singularities of the corresponding univariate GF play an important role. In Poznanovic and Heitsch (2014), the authors studied this very GF, so we cite those results when necessary here.

#### Proposition 7

Define ρ as the unique smallest positive solution to $R(z) = (1 - p_1q_2z)^2(1-p_3z^2) - 4p_2q_1q_2q_3z^3$, and define $\delta = p_1q_2\rho$. Consider the distribution assigned by the Pfold grammar on dot-bracket sequences $\mathcal {L}$ as their length $n \rightarrow \infty$. Then, the joint limiting distribution of DEG and UNP is given by the bivariate PGF,

$$ \frac{(1-\delta )^2v}{(1 + \delta + (\delta ^2 - \delta )v - (\delta ^2 + \delta )u)^2}. $$

From this, the limiting distributions of DEG, UNP, CHN, LEN, and ETE are as in Table 2.

#### Proof

We first need to rewrite the Pfold grammar so that DEG and UNP can be more easily tracked. To do this, we add two additional symbols, $S_{{{\,\textrm{ext}\,}}}$ and $L_{{{\,\textrm{ext}\,}}}$, corresponding to when the grammar is still producing part of the exterior loop. Then, $S_{{{\,\textrm{ext}\,}}}$ becomes the new start symbol for the grammar, and we can mark when the grammar transitions from the exterior loop to other nucleotides by dropping the “${{\,\textrm{ext}\,}}$” tag. We add the following two rules:

$$ [1']\ S_{{{\,\textrm{ext}\,}}} \rightarrow \ L_{{{\,\textrm{ext}\,}}}S_{{{\,\textrm{ext}\,}}} \ (p_1)\ \big |\ L_{{{\,\textrm{ext}\,}}} \ (q_1) \hspace{0.4 in} [2']\ L_{{{\,\textrm{ext}\,}}} \rightarrow (F) \ (p_2) \ \big |\ .\ (q_2) $$

Importantly, these rules still use the same probabilities as rules [1] and [2] in the original grammar so that the distribution of structures remains unchanged.

By using the Chomsky–Schützenberger theorem, we can convert this recursive definition of the grammar’s symbols into a system of equations for the trivariate generating function $S_{{{\,\textrm{ext}\,}}}(z, u, v) = \sum p(n, k, \ell ) z^n u^k v^\ell$ where p(n,k,ℓ) represents the probability that the grammar outputs a structure of length *n* with *k* unpaired nucleotides and degree ℓ:

$$\begin{aligned} &  [1] \ S(z, u, v) = p_1 LS + q_1L \quad \qquad [2]\ L(z, u, v) = p_2z^2F + q_2z \quad \qquad [3]\ F(z, u, v) \\ &  \quad = p_3z^2F + q_3LS \end{aligned}$$

$$ {[}1']\ S_{{{\,\textrm{ext}\,}}}(z, u, v) = p_1 L_{{{\,\textrm{ext}\,}}}S_{{{\,\textrm{ext}\,}}} + q_1L_{{{\,\textrm{ext}\,}}} \hspace{.4 in} [2']\ L_{{{\,\textrm{ext}\,}}}(z, u, v) = p_2vz^2F + q_2uz $$

The functions *S*, *L*, *F*,  and $L_{{{\,\textrm{ext}\,}}}$ are auxiliary GFs that correspond to the output of the grammar had it started with the corresponding symbol instead of $S_{{{\,\textrm{ext}\,}}}$, but they are useful in solving for $S_{{{\,\textrm{ext}\,}}}$. One way to find the minimal polynomial for $S_{{{\,\textrm{ext}\,}}}$ is to use Gröbner bases, as illustrated in our companion SageMath worksheet. Overall, we obtain a function of the form

5 $$\begin{aligned} S_{{{\,\textrm{ext}\,}}}(z, u, v) = \frac{-b(z, u, v) - q_1v\sqrt{(1-z^2p_3)R(z)}}{2a(z, u, v)}. \end{aligned}$$

Recall that $R(z) = (1 - p_1q_2z)^2(1-p_3z^2) - 4p_2q_1q_2q_3z^3$, which is the function determining the singularities of the univariate GF tracking only the length of the output of the grammar. From Poznanovic and Heitsch (2014, Lemma 3.3), the unique minimal zero ρ of *R*(*z*) has $\rho < 1/\sqrt{p_3}$, so z=ρ is indeed the minimal singularity of the function underneath the square root.

There are two technically challenging components remaining to finish deriving the limiting PGF. First, we must show that z=ρ is the unique minimal singularity for $S_{{{\,\textrm{ext}\,}}}(z, u, v)$ for all *u* and *v* sufficiently small. Then, the expression for *a*(*z*, *u*, *v*) is exceedingly messy. To recognize the limiting distribution as related to negative binomials, we must find a way to rewrite it.

For the first challenge, we find that the singularities of $S_{{{\,\textrm{ext}\,}}}(z, u, v)$ either come from the zeroes of *a*(*z*, *u*, *v*) or $(1-z^2p_3)R(z)$. Having analyzed the latter already, we look at the zeroes of *a* when *u* and *v* are small. The zeroes of *a*(*z*, 0, 0) are of magnitude $z = \sqrt{p_1/(p_1p_3 - p_2q_1q_3)}$, as verified in the SageMath worksheet. It is easy to see that $\sqrt{1/p_3}$ is strictly smaller than this, and Poznanovic and Heitsch (2014, Lemma 3.3) again verifies that $\rho < \sqrt{1/p_3}$. Hence, |*a*(*z*, 0, 0)| is bounded away from zero for all $|z| \le \rho$. Because *a*(*z*, *u*, *v*) is a polynomial, for *u* and *v* sufficiently small, |*a*(*z*, *u*, *v*)| is also bounded away from zero for all $|z| \le \rho$. Hence, we have satisfied the conditions of Proposition 2 and can compute the limiting probability distribution.

Now, to rewrite *a*(*z*, *u*, *v*) in a more convenient form, it is useful to note that *a*(*z*, *u*, *v*) will only be evaluated when z=ρ. So, we are free to add and subtract multiples of *R*(*z*) without changing the output of the proposition. By matching terms by hand, we find:

$$\begin{aligned} 4q_2z \cdot a(z, u, v)= &  (p_1^2q_2^2uz^2 - p_1^2q_2^2vz^2 + p_1q_2uz + p_1q2vz - p_1q_2z - 1)^2\\ &  (p_3z^2 - 1) + C(z, u, v)R(z) \end{aligned}$$

for an appropriate choice of *C*(*z*, *u*, *v*) given in the SageMath worksheet. Substituting this form of *a*(*z*, *u*, *v*) into Proposition 2 with $g(u, v) = q_1v$ yields the limiting PGF given in the proposition statement.

The default values of the $p_i$ are given in the original paper (Knudsen and Hein 2003) as $p_1 = 0.868534$, $p_2 = 0.105397$, and $p_3 = 0.787640$. This leads to the corresponding singularity ρ=1.000173 and a default δ value of δ=0.777127, as calculated in our SageMath worksheet. Following the same steps as for the Dyck and Motzkin paths, we can find the distributions of each parameter in the proposition with appropriate substitutions or series expansions of this PGF. □

## The Limiting Distributions in Focus

Between Lemma 3, Corollary 6, and Proposition 7, we identified the limiting distributions of various characteristics of end-to-end distance among our models, as summarized in Table 2. But, what do these distributions say about end-to-end distance?

Every parameter we studied stabilizes into a length-independent distribution with a finite mean. This is implied by the fact that every limiting distribution is discrete. This means that as the length of a sequence increases, we do not expect these parameters to grow without bound. Below, we visualize the distributions and compare them between models.

The main punchline is that every distribution we have encountered has a short mean and light tails. In fact, any negative binomial distribution *N*(*k*, *p*) with 0<p<1 has exponentially decaying tails. Moreover, this immediately implies that the limiting distribution of ETE has tails decaying faster than exponentially: see Lemma 8 below. Therefore, each parameter is concentrated near its mean and has finite moments of all orders.

A normal distribution has exponential tails of the form $e^{-x^2}$, which indicates the tails here are asymptotically heavier than the normal distribution for large values of each parameter. Nonetheless, when compared to the normal distribution, the negative binomial distributions for DEG have a higher proportion within one standard deviation of the mean, as described in Sect. 1.

**Fig. 3:**
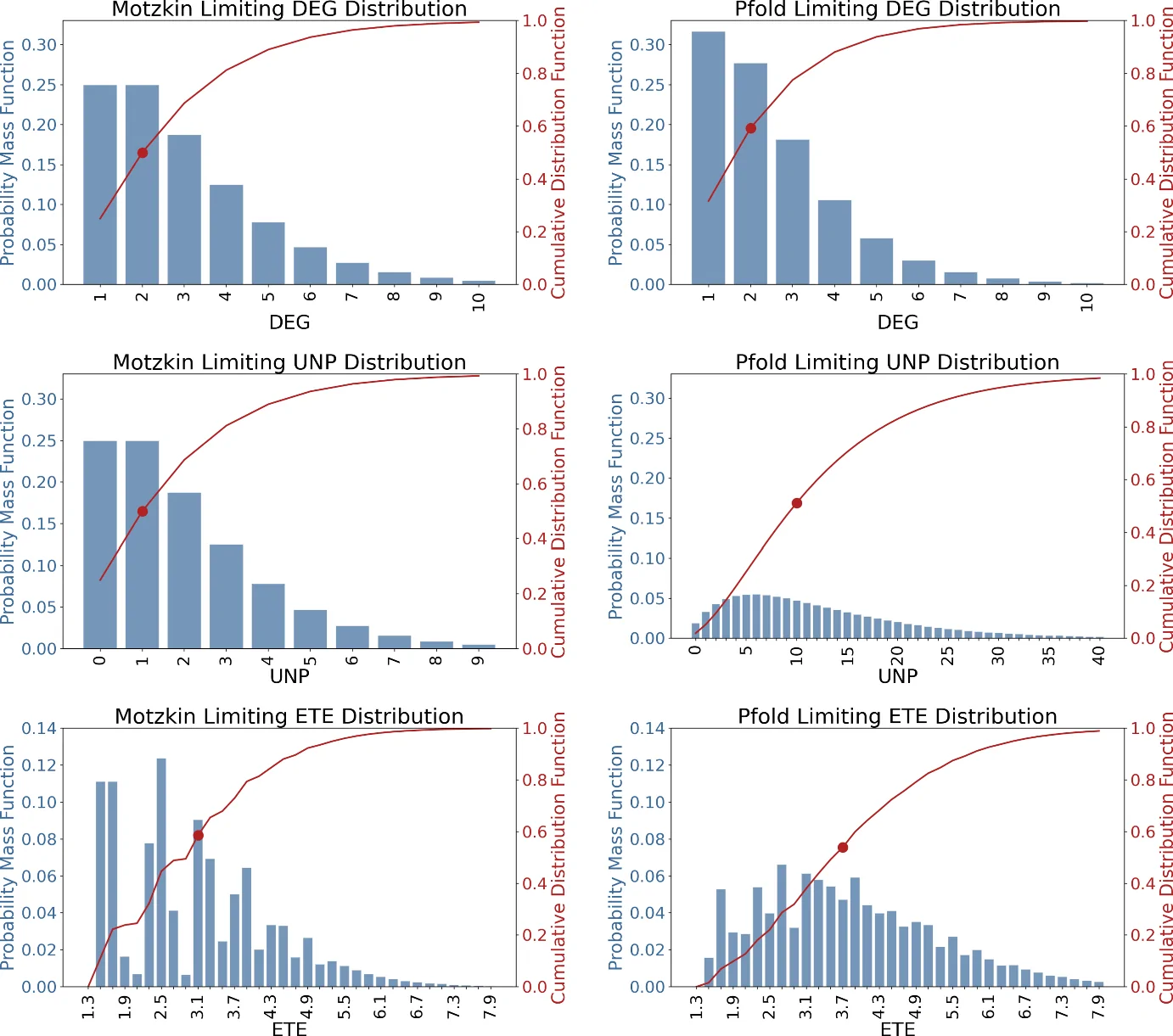
On each graph, the left axis represents the probability mass function, and the right axis represents the cumulative distribution function. The median is indicated by a red dot. Left column: The limiting distributions for the Dyck and Motzkin path models. Right: The limiting distributions for the Pfold grammar with default parameters (Color figure online)

In the formula for ETE (Eq. 1), there are two components corresponding to the bond types in the exterior loop: CHN corresponding to covalent links, and DEG corresponding to hydrogen bridges between base pairs. Since $\texttt {CHN}= \texttt {DEG}+ \texttt {UNP}- 1$, we can also reframe ETE in terms of only DEG and UNP. In the sections below, we examine the limiting distributions of DEG and UNP, and explore their impact on the limiting distribution of ETE. Figure 3 illustrates the limiting distributions for each of these parameters under our models of secondary structures.

### Limiting DEG Distributions

The mean for DEG is uniformly low across the Dyck paths, Motzkin paths, and Pfold grammar. Because the distribution is consistently a negative binomial across all models, the tails of the distributions are bounded by exponential functions. But, the Pfold grammar more heavily weights low degree structures, which lowers the mean from 3 to 2.55. We give a combinatorial argument for why the distributions of DEG are identical for Dyck and Motzkin paths in Appendix A.

We can measure how much more concentrated the Pfold DEG distribution is by exploring the tails of the distributions. For the Dyck and Motzkin path distributions, $\mathbb {P}(\texttt {DEG}> 5) = 0.11$, while for the Pfold grammar, $\mathbb {P}(\texttt {DEG}> 5) = 0.06$. This implies that high degree outliers are much less common under the Pfold model.

### Limiting UNP Distributions

Although the distributions of UNP are also negative binomials, there is a much larger difference between the Motzkin UNP distribution and the Pfold UNP distribution. For Motzkin paths, UNP is generally even smaller than DEG, with a mean of only 2. Under this model, there is a 50% chance that a secondary structure has zero or one unpaired nucleotides. In contrast, for the Pfold grammar, UNP is generally much larger than DEG, with a mean of 12.39. Here, there is only a 5% chance that a secondary structure has zero or one unpaired nucleotides.

To emphasize how separated the distributions are, we note that $\mathbb {P}(\texttt {UNP}> 5) = 0.06$ for the Motzkin unpaired distribution, but $\mathbb {P}(\texttt {UNP}> 5) = 0.75$ for the Pfold unpaired distribution. Although the mean of the Pfold unpaired distribution is much higher, the tails ultimately are still bounded by an exponential decay.

### Limiting ETE Distributions

Evaluating the limiting distribution of ETE in our models is difficult because DEG and UNP are not independent. In fact, for the Motzkin path model, the correlation of DEG and UNP is 0.5. Upon reflection, this is hardly surprising because the more times a Motzkin path returns to the horizon y=0, the more likely it is to have horizontal steps on the horizon. For the Pfold grammar with default parameters, this correlation increases to 0.61.

Because of this lack of independence, it is crucial to track the limiting joint distribution of DEG and UNP simultaneously in order to assess the limiting distribution of ETE. Unlike CHN and LEN which are linear combinations of DEG and UNP, the fact that the equation for ETE is non-linear (Eq. 1) implies that the mean for ETE cannot be expressed in terms of the means of DEG and UNP alone. However, by using multivariate generating functions that track both DEG and UNP simultaneously, we are able to compute any moment of ETE to arbitrary precision and also plot the probability mass function, shown in the bottom panels in Fig. 3.

**Fig. 4:**
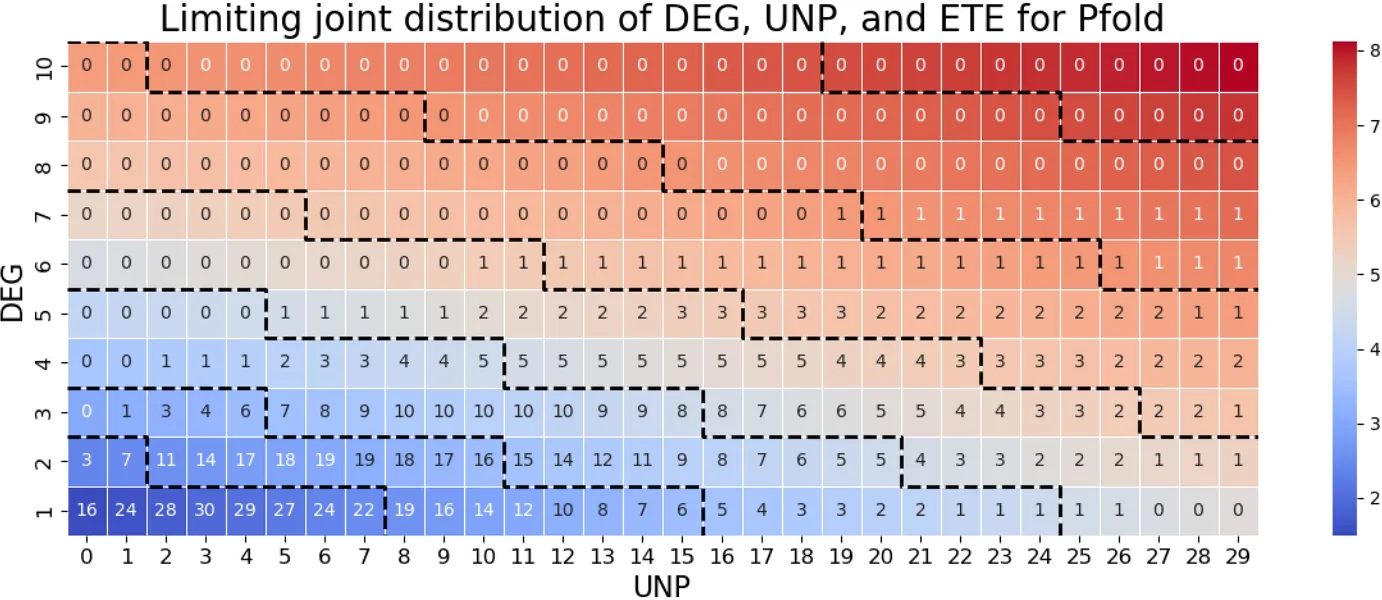
The joint distribution of DEG and UNP for the Pfold grammar with default parameters. The numbers in each cell represent the expected number of structures with a given pair of $(\texttt {DEG}, \texttt {UNP})$ values out of a sample of size 1000. The dashed black lines separate ETE values to the nearest integer. For instance, the lowest left block represents ETE values that round to 2, and crossing the nearest dashed line transitions to ETE values that round to 3 (Color figure online)

In the limiting ETE distributions for Motzkin paths and the Pfold grammar, the distributions begin with concentrated blocks that give a jagged appearance. This happens for two reasons: first, there are relatively few combinations of DEG and UNP that lead to small ETE values. This is visualized in the Pfold joint distribution in Fig. 4, where the dashed lines separate blocks by ETE value, rounded to the nearest integer. Secondly, a change in DEG impacts the value of ETE much more than a change in UNP.

For the Motzkin limiting distribution, UNP is rarely ever large. Thus, the spikes in the graph correspond to increasing degrees, and the spikes are particularly prominent. In contrast, for the Pfold limiting distribution, UNP covers a much larger range of values, meaning that the blocks are less jagged. In both models, as ETE values increase, there are more possible $(\texttt {DEG}, \texttt {UNP})$ combinations that could contribute to a given block, but the corresponding probabilities decay super-exponentially (as we will soon prove). This leads to the smoother super-exponential tails in the bottom graphs of Fig. 3.

#### Lemma 8

Under the Motzkin model and the Pfold grammar, ETE has tails that decay super-exponentially.

#### Proof

Recall the formula for ETE,

$$ \texttt {ETE}= \sqrt{b^2 \cdot \texttt {DEG}^{6/5} + c^2 \cdot \texttt {CHN}^{6/5}}, $$

with b=1.5 and c=0.62. Note that if $\texttt {ETE}> t$, then at least one of the following must be true: $\texttt {DEG}> (t/2b)^{5/3}$ or $\texttt {CHN}> (t/2c)^{5/3}$. Otherwise, from the triangle inequality,

$$ \texttt {ETE}\le b \texttt {DEG}^{3/5} + c \texttt {CHN}^{3/5} \le t. $$

Any negative binomial random variable *X* with distribution *N*(2, *p*) with 0<p<1 has tails that can be bounded by an exponential,

$$ \mathbb {P}(X > t) \le Ce^{-kt}, $$

for some C,k>0. This could be verified directly by summing the probabilities $\mathbb {P}(X = \ell )$ for ℓ>t and using common series summation formulas. Therefore, for appropriate constants *C* and *k*,

$$\begin{aligned} \mathbb {P}(\texttt {ETE}> t)&\le \mathbb {P}(\texttt {DEG}> (t/2b)^{5/3}) + \mathbb {P}(\texttt {CHN}> (t/2c)^{5/3})\\&\le C_1e^{-k_1t^{5/3}} + C_2 e^{-k_2t^{5/3}}\\&\le C_3e^{-k_3t^{5/3}}. \end{aligned}$$

Hence, $\lim _{t \rightarrow \infty } \mathbb {P}(\texttt {ETE}> t)/(Ce^{-kt}) = 0$ for any constants C,k>0, so by definition, the tails on ETE decay super-exponentially. □

## Assessing End-Proximity in mRNA and lncRNA

As remarked in Sect. 1, the end-proximity consistently identified in rna molecules is viewed as surprising when compared to the expected end-to-end distance for a random linear polymer. However, our theoretical results here are for *branched* structures formed from linear polymers under noncrossing self-pairings. For all three models considered, we prove that the limiting ETE has small mean, as well as moderate variance due to the super-exponential decay of the tails. In other words, irrespective of sequence length or nucleotide content, it would be much more surprising to find that the ends of an rna molecule *aren’t* close than to see that they are.

But this then raises the dichotomy posed in our title. Are rna molecules “making ends meet” or simply “meeting at the ends”? We now formulate the distinction more precisely by identifying the former with the Pfold distribution on dot-bracket sequences and the latter with the Motzkin path one since the difference is designed^3^ to capture characteristics of rna base pairing.

Intuitively, under the rna-like, i.e. biological, grammar parameters, the probability that a helix is initiated is low ($p_2 \sim 0.11$), but the likelihood that it continues is high ($p_3 \sim 0.79$). As a consequence, the DEG distribution shifts slightly lower, but the UNP is markedly higher. However, the resulting effect on ETE is unlikely to be experimentally detectable; a 6-fold increase in UNP mean only increases the physical distance estimate by 0.75 nm, which is well within the measurement error (Lai et al. 2018).

Hence, to address our question, we must consider not only the overall ETE estimate, but also the distributions of the constituent end-proximity characteristics. These cannot be measured experimentally, but rather must be determined from the inferred base pairings. In this way, we are able to show that the 10 sequences^4^ from Lai et al. (2018) listed in Table 3 are more similar to the Pfold distributions than the Motzkin ones. Thus, they are indeed meeting at the ends.

**Table 3:** The 8 mrna and 2 lncrna sequences from Lai et al. (2018) and their lengths, which differ by an order of magnitude

Abbreviation	Length	Sequence
RPL41A	327	Yeast RPL41A mrna
ATP	424	Human ATP5J2 mrna
HSBP1	535	HSBP1 mrna
MIF	562	MIF mrna
Bglobin	590	Rabbit β-globin mrna
MRP51	674	MRPL51 mrna
GAPDH	1327	GAPDH mrna
FLuciferase	1658	Firefly luciferase mrna
Hotair	2148	HOTAIR lncrna
Neat1	3734	NEAT1_S lncrna

Because our secondary structures will be generated by thermodynamic optimization, we compare the “natural” characteristics not only to the theoretical ones but also to a suitable “artificial” background. In this way, we confirm whether and how much any differences from the models are a general reflection of the NNTM objective function rather than a more specific biological signal.

**Fig. 5:**
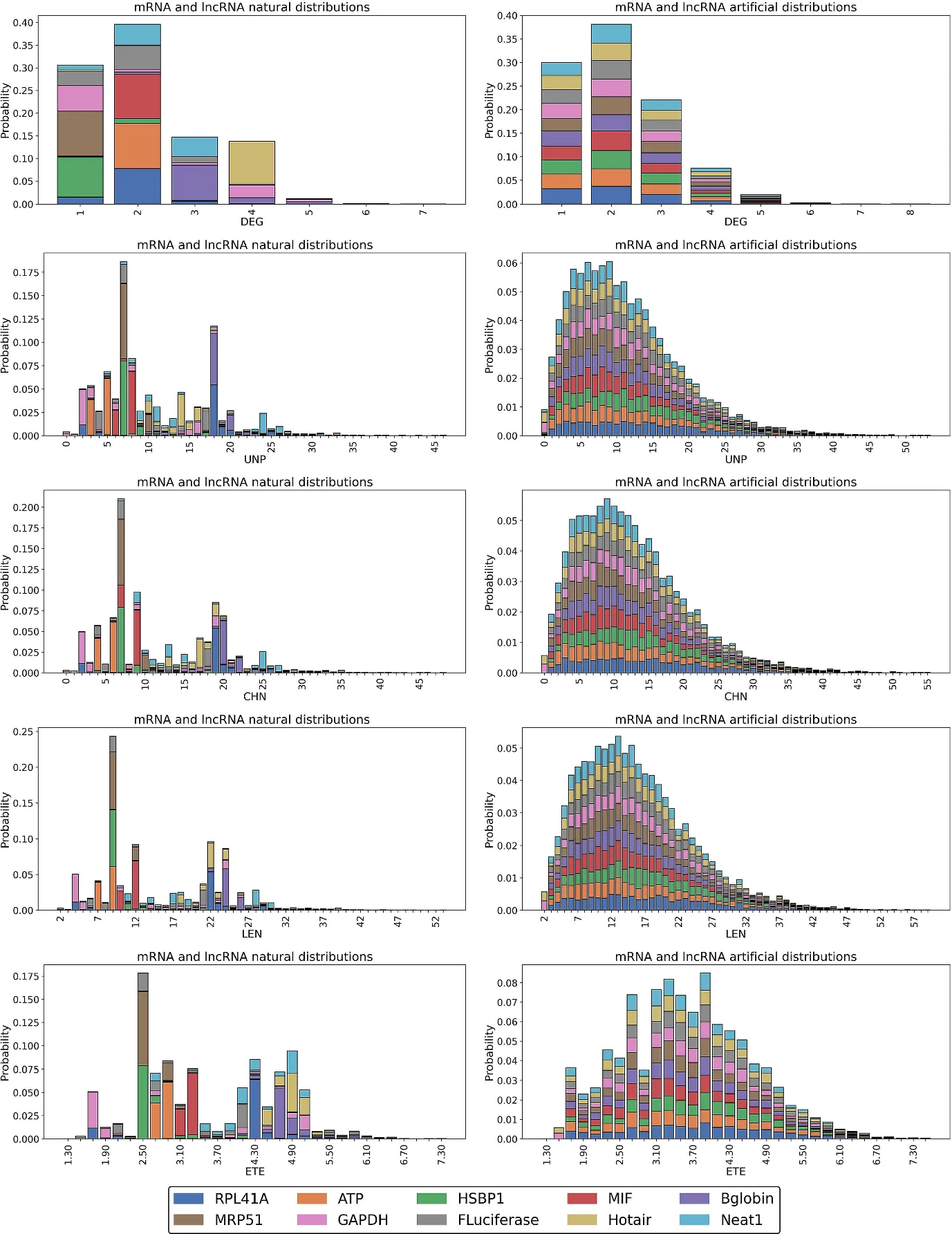
Each of the distributions of end proximity for the mrna and lncrna sequences studied in Lai et al. (2018). On the left, samples of 1000 structures generated using stochastic for each of the sequences. On the right, each sequence was shuffled 1000 times and the MFE structure was found using RNAfold. Then, each of the end proximity parameters were measured for these MFE structures (Color figure online)

For every sequence, we consider two different samples of 1000 secondary structures each, generated as described below. We compute the end-proximity characteristics for each structure and report averages and standard deviations, aggregated by sequence and by sample type, in Table 4. We also consider the overall distributions pictured in Fig. 5, and discuss comparisons within and between the two sets of structures as well as with our theoretical distributions.

**Table 4:** Distribution statistics for mrna and lncrna natural distributions, the Pfold and Motzkin theoretical distributions, and then the mrna and lncrna artificial distributions

Seq.	DEG		UNP		LEN		CHN		ETE	
	μ	σ	μ	σ	μ	σ	μ	σ	μ	σ
*mRNA and lncRNA natural distributions*										
RPL41A	1.9	0.5	16.6	6.8	20.5	7.6	17.6	7.2	4.0	1.0
ATP	2.0	0.1	4.3	1.1	8.3	1.0	5.3	1.0	2.8	0.1
HSBP1	1.1	0.4	7.8	2.5	10.0	2.9	7.9	2.7	2.7	0.4
MIF	2.0	0.2	7.5	1.5	11.5	1.7	8.5	1.6	3.2	0.2
Bglobin	3.2	0.6	18.0	4.2	24.3	4.5	20.2	4.3	4.8	0.5
MRP51	1.0	0.2	7.6	1.6	9.7	1.8	7.7	1.7	2.6	0.2
GAPDH	2.2	1.5	7.4	6.7	11.8	9.1	8.6	7.9	3.1	1.5
FLucif	1.9	0.8	12.3	8.1	16.2	9.3	13.3	8.7	3.6	1.1
Hotair	3.9	0.6	13.5	3.4	21.2	4.2	16.4	3.8	4.7	0.6
Neat1	2.3	0.7	15.7	7.3	20.4	7.1	17.0	7.2	4.2	0.8
Combined	2.2	1.1	11.1	6.8	15.4	8.0	12.2	7.3	3.6	1.1
*Theoretical distributions*										
Pfold	2.6	1.7	12.4	4.3	17.5	11.8	14.0	10.5	3.8	1.5
Motzkin	3.0	2.0	2.0	2.0	8.0	5.3	4.0	3.5	3.1	1.2
*mRNA and lncRNA artificial distributions*										
Combined	2.1	1.0	11.2	7.4	15.5	8.2	12.4	7.7	3.6	1.0
RPL41A	2.1	1.0	13.4	8.6	17.6	9.4	14.5	8.9	3.8	1.1
ATP	2.2	1.0	11.7	8.4	16.0	9.2	12.9	8.2	3.6	1.1
HSBP1	2.1	1.0	12.6	7.4	16.9	8.2	13.7	7.8	3.7	1.0
MIF	2.1	1.0	9.9	6.2	14.1	7.0	11.0	6.5	3.4	0.9
Bglobin	2.2	1.1	10.4	6.2	14.7	7.0	11.6	6.5	3.5	0.9
MRP51	2.2	1.0	9.8	6.2	14.3	7.0	11.1	6.5	3.5	0.9
GAPDH	2.1	1.0	11.0	7.3	15.2	8.1	12.1	7.6	3.5	1.0
FLucif	2.1	1.0	12.2	7.9	16.4	8.6	13.3	8.2	3.7	1.0
Hotair	2.2	1.0	11.3	7.9	15.6	8.8	12.4	8.3	3.6	1.1
Neat1	2.2	1.1	10.1	6.4	14.5	7.3	11.3	6.7	3.5	1.0

Each sequence was shuffled 1000 times

The *artificial* set is generated by randomly permuting each sequence 1000 times using uShuffle (Jiang et al. 2008) while maintaining 2-let counts, and then using RNAfold from the ViennaRNA software suite (Lorenz et al. 2011) to predict the MFE secondary structure for each shuffle. Hence, these sequences have the same length and dinucleotide content as the natural counterpart, but no biological function. Thus, their characteristics provide a sequence-specific background distribution under thermodynamic optimization containing no biological information other than free energy minimization.

In contrast, the *natural* set is generated by sampling structures from the Boltzmann ensemble using the stochastic program from the RNAstructure software suite (Reuter and Mathews 2010). This method of predicting the end-to-end distance was validated against experimental data (Lai et al. 2018) for the 10 sequences. In conjunction with a two-scale freely jointed chain approximation (Aalberts and Nandagopal 2010), they found that the predicted average ETE distance^5^ correlates with their measurements from Förster resonance energy transfer (FRET) experiments. They demonstrated that, contrary to expectations for mrna and lncrna, protein factors are not necessary for small ETE. Rather they emphasize the importance of the intramolecular basepairing interactions in producing rna secondary structures with short end-to-end distances.

They found that the ETE values from experimental measurements were always ≤7 nm, and that the computational predictions for a large number of human mrna and lncrna were narrowly distributed with a mean of $\sim \,4$ nm and almost none >8 nm. Although these are properties held by the Pfold grammar, they are not sufficiently dissimilar from the Motzkin path distributions to distinguish between the two.

More specifically, the overall ETE summary statistics are nearly identical between the natural and artificial sets. Although they are closer to the Pfold than the Motzkin ones, the differences would not be significant over the experimental sample sizes (i.e. of 3–5 trials per sequence). A critical distinction, however, is that the individual averages and standard deviations for the artificial ETE are narrowly grouped around the overall mean, whereas the natural ones are more broadly distributed. While this allows us to distinguish some individual natural distributions from others, e.g. Bglobin from MRP51, neither are distinguishable from the two theoretical distributions which both fall between the extreme values.

Unpacking the components of the ETE formula, similar conclusions apply at first. The combined summary statistics for DEG, and also for UNP, are essentially the same between the natural and artificial sets, although the latter are again quite narrowly distributed with higher variability while the former are more spread out with generally smaller standard deviations.

Now though a clearer distinction in comparison with the Pfold and Motzkin distributions begins to emerge. From the artificial sequences, we understand that the expected degree distribution under the NNTM optimization is lower than the Pfold one, and considerably lower than the Motzkin. In the natural sequences, we see deviations on both the low (HSBP1, MRP51) and high (Bglobin, Hotair) ends. But only the high ones might be candidates for “meeting at the ends.” However, all the UNP distributions except for ATP are very far from the Motzkin one, although not dissimilar to Pfold. Hence, we conclude that these rna sequences are indeed “making ends meet.”

We now focus on differences in the way that is achieved by considering the full distributions in Fig. 5. In particular, we see that the artificial ETE distribution closely resembles the Pfold one, with spikes as described in Sect. 4.3, while the natural one is quite different. The key difference is in the concentration of the natural values versus the dispersal of the artificial ones for individual sequences.

Over a 10-fold length difference, the artificial sequences exhibit essentially the same end-proximity characteristics, and their distributions closely resemble the Pfold theoretical ones. In contrast, although the natural characteristics are also distributed around the Pfold ones, we see that they are coming from distinct structural modes. The DEG values are highly concentrated, apparent in the many low per-sequence standard deviations (with the exception of GAPDH), but range from l.1 to 3.9. As expected from the theoretical results, there is a corresponding difference in those ETE values of 2.7 and 4.7.

Interestingly, however, the UNP values can play a much more prominent role in the ETE determination than expected from the theoretical analysis. Although RPL41A has the third smallest DEG (at 1.9, tied with FLucif), its large UNP value of 16.6 results in the fourth largest ETE at 4.0. From this we understand that not only is the concentration of degree a clear biological signal, but a particularly large (or small like ATP) number of unpaired bases relative to the degree can be too.

In the next section, we consider how the distributions of DEG, UNP, and ETE for the mrna/lncrna compare to other types of rna molecules, specifically those whose secondary structure is known through methods other than thermodynamic optimization.

## Assessing ETE in Known RNA Secondary Structures

**Table 5:** The first five families are from the ArchiveII dataset (Sloma and Mathews 2016; Mathews 2019), while the ssu rrna and lsu rrna sequences are from the Comparative RNA Web-2 website (Cannone et al. 2002; Chan et al. 2023)

Family	tRNA	SRP	5 S	RNaseP	tmRNA	ssu	lsu
Median length	76	118	119	330	363	1509	3021
#Sequences	557	924	1283	454	462	54	38

Along the spectrum in Fig. 1, we have shown that the ends of branched structures are necessarily close — independent of sequence length, nucleotide content, and even base pairing model. From Dyck paths through Motzkin ones to the Pfold grammar, the difference in expected ETE is within experimental FRET error (Lai et al. 2018).

Moreover, for a diverse set of mrna and lncrna, both natural and artificial computational predictions yield overall end-proximity summary statistics which are indistinguishable from each other. In particular, whether narrowly (artificial) or broadly (natural) grouped around the combined mean, the expected end-to-end distance is $\sim \,4$ nm and certainly ≤7 nm.

However, we have seen that while the overall artificial distributions closely resemble the Pfold theoretical ones, the distribution for the natural characteristics was typically much more concentrated, with clear differences for each sequence.

We now turn our attention to other types of rna molecules whose distributions we expect to be even more concentrated. Specifically, we complete our analysis spectrum by examining rna whose base pairings are considered known through comparative sequences alignment and/or other methods beyond just free energy minimization. In particular, we analyze five families from the ArchiveII dataset (Sloma and Mathews 2016; Mathews 2019) and a curated selection of 16 S and 23 S ribosomal rna from the Comparative RNA Web-2 website (Cannone et al. 2002; Chan et al. 2023). Further details on these datasets are given in Table 5 and the subsections below.

We again compare the natural distributions against their artificial counterparts and the theoretical ones. Now, however, the natural characteristics are evaluated from the known secondary structures for each family while the artificial ones come from an MFE prediction for five different dinucleotide shuffles for each sequence in the family. The summary statistics for the ArchiveII end-proximity characteristics are given in Table 6, and the ribosomal ones in Table 7. Figures with the overall distributions appear in Appendix C.

### ArchiveII: Functional Molecules with Shorter Sequences

**Table 6:** Distribution statistics for natural distributions from the ArchiveII dataset, the Pfold and Motzkin theoretical distributions, and the artificial ArchiveII distributions

Seq.	DEG		UNP		LEN		CHN		ETE	
	μ	σ	μ	σ	μ	σ	μ	σ	μ	σ
*ArchiveII natural distributions*										
trna	1.0	0.0	4.0	0.6	6.0	0.6	4.0	0.6	2.1	0.1
SRP rna	1.5	0.7	6.2	5.0	9.3	5.6	6.8	5.3	2.7	0.8
5 S rrna	1.0	0.1	2.0	2.1	4.0	2.1	2.0	2.1	1.8	0.3
RNaseP	1.2	0.6	6.4	4.1	8.8	5.0	6.6	4.5	2.5	0.7
tmrna	1.0	0.2	3.9	1.0	5.9	1.2	3.9	1.1	2.1	0.2
Combined	1.2	0.5	4.2	3.7	6.5	4.2	4.3	3.9	2.2	0.6
*Theoretical distributions*										
Pfold	2.6	1.7	12.4	4.3	17.5	11.8	14.0	10.5	3.8	1.5
Motzkin	3.0	2.0	2.0	2.0	8.0	5.3	4.0	3.5	3.1	1.2
*ArchiveII artificial distributions*										
Combined	1.9	0.9	10.0	6.6	13.9	7.2	11.0	6.9	3.4	0.9
trna	1.7	0.7	9.9	6.8	13.4	7.0	10.7	6.9	3.3	0.9
SRP rna	1.9	0.9	9.2	6.0	13.1	6.5	10.2	6.2	3.3	0.9
5 S rrna	2.0	0.9	10.2	6.3	14.1	6.9	11.1	6.6	3.4	0.9
RNaseP	2.1	1.0	10.4	7.2	14.5	7.9	11.5	7.5	3.5	1.0
tmrna	2.1	1.0	11.1	7.6	15.4	8.4	12.2	7.9	3.6	1.1

Each sequence was shuffled 5 times

As expected (Ermolenko and Mathews 2020), we clearly see the evolutionary pressure on the exterior loop for these families; the transfer rna (trna), 5 S ribosomal rna (rrna), and transfer-messenger rna (tmrna) sequences exhibit particularly strong homology in having a single helix and a small range of unpaired nucleotides. This results in ETE values which are distinctly lower than any others, and more than a standard deviation from the Pfold average (although not the Motzkin one). In comparison, signal recognition particle (SRP) rna and Ribonuclease P (RNaseP) have slightly higher ETE averages and standard deviations, although still among the lowest seen.

**Fig. 6:**
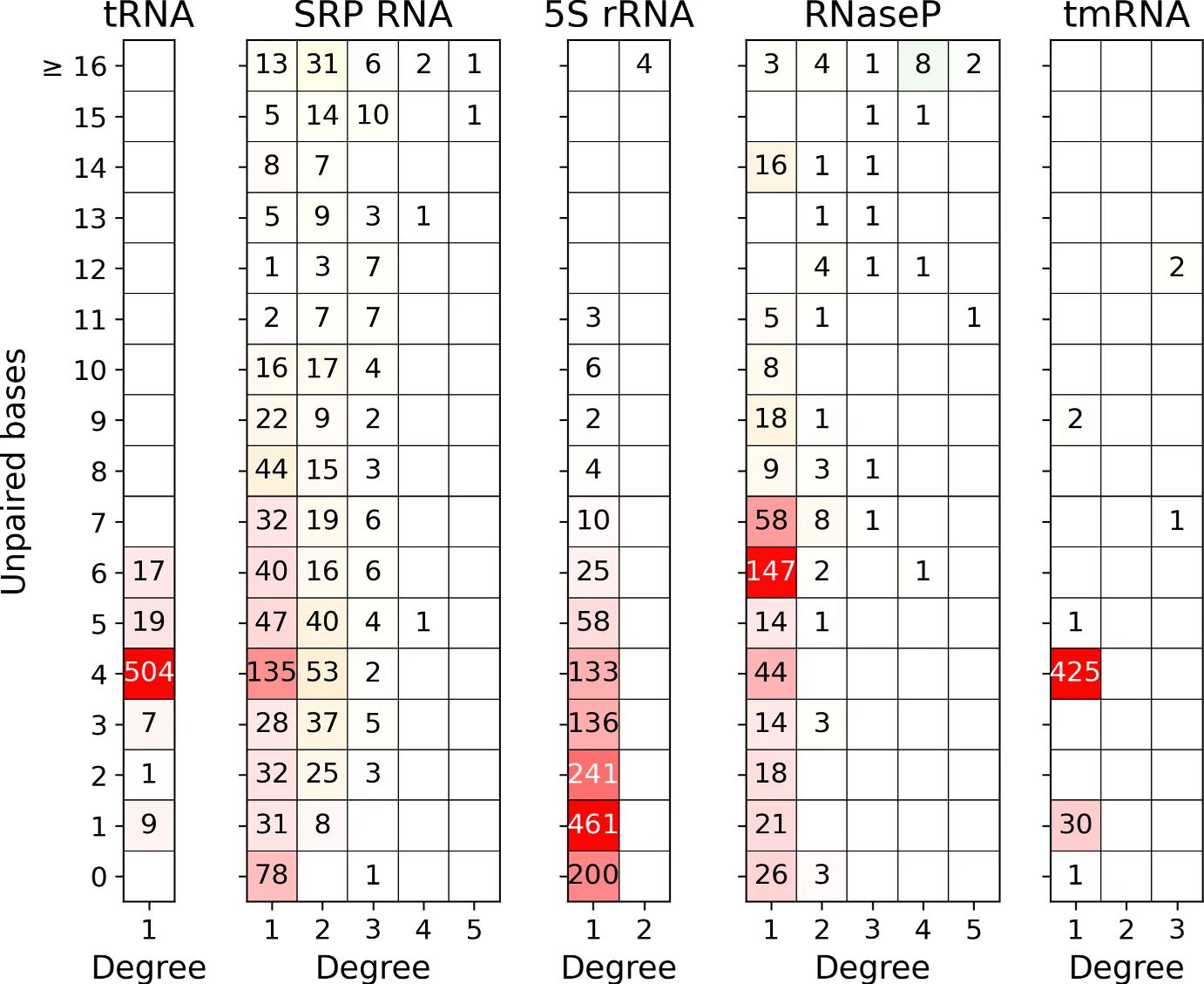
The distribution of DEG versus UNP across the ArchiveII dataset. The intensity of each color represents the percent of structures with the corresponding DEG and UNP values. Colors represent approximate ETE distances, color-coded as red: 1.5–2.5, orange: 2.5−3.5, yellow: 3.5−4.5, green: 4.5−5.5, blue: 5.5−6.5, purple: 6.5−7.5 (Color figure online)

To explore this additional variability, we visualize the joint distribution of DEG and UNP across each ArchiveII family in Fig. 6. For RNaseP, DEG remains concentrated at 1 (88%), but there is more variability in the UNP values. Although the majority (58%) of SRP rna sequences also have $\texttt {DEG}= 1$, 34% have $\texttt {DEG}= 2$ and 7% even have $\texttt {DEG}= 3$, with all degrees exhibiting a broad range of UNP values.

We note the variability observed in UNP for RNaseP, and in both DEG and UNP for SRP rna, is not inconsistent with the different secondary structure visualizations available in Rfam (Griffiths-Jones 2003). However, some of the smaller fluctuations may also be a result of the ArchiveII analysis. When a secondary structure is inferred from a sequence alignment or other comparative methods, a region with a high percentage of unpaired bases is more likely to be an absence of pairing information rather than a positive signal for single-strandedness. To compensate for this, the ArchiveII structure was used as a constraint for an RNAfold prediction whose exterior loop was then analyzed.

The highly concentrated degree, and generally narrow range of UNP values, strongly suggest that the trna, 5 S rrna, tmrna and RNaseP sequences are doing more than “making ends meet.” Their ends are, in fact, “bound to meet” particularly in comparison with their artificial counterparts. The end-proximity characteristics for the MFE shuffles much more closely resemble the mrna/lncrna, and by extension the Pfold, ones, although the shorter sequence lengths in the ArchiveII families seem to be reflected in slightly lower values overall.

### CRW-2: Ribosomal RNA with Longer Sequences

**Table 7:** Distribution statistics for natural distributions for the ribosomal rrna, the Pfold and Motzkin theoretical distributions, and the ribosomal artificial distributions

Seq.	DEG		UNP		LEN		CHN		ETE	
	μ	σ	μ	σ	μ	σ	μ	σ	μ	σ
*SSU rRNA and LSU rRNA natural distributions*										
ssu	4.0	0.0	36.4	11.3	44.4	11.3	39.4	11.3	6.6	0.8
lsu (Combined)	4.4	4.5	24.3	35.2	33.0	42.4	27.6	38.7	4.9	3.9
Archaea	2.4	3.5	12.7	23.4	17.6	30.3	14.1	26.8	3.2	3.1
Bacteria	1.0	0.0	2.3	0.6	4.3	0.6	2.3	0.6	1.8	0.1
Eukarya	7.6	4.2	44.6	39.8	59.7	45.2	51.2	42.4	7.7	3.7
Combined	4.2	2.9	31.4	24.9	39.7	29.2	34.5	27.0	5.9	2.7
*Theoretical distributions*										
Pfold	2.6	1.7	12.4	4.3	17.5	11.8	14.0	10.5	3.8	1.5
Motzkin	3.0	2.0	2.0	2.0	8.0	5.3	4.0	3.5	3.1	1.2
*SSU rRNA and LSU rRNA artificial distributions*										
Combined	2.2	1.0	11.7	7.4	16.0	8.2	12.9	7.7	3.7	1.0
ssu	2.2	1.0	11.6	7.4	16.0	8.1	12.8	7.7	3.7	1.0
lsu (Combined)	2.2	1.0	11.7	7.4	16.1	8.2	12.9	7.8	3.7	1.0
Archaea	2.1	1.1	9.9	6.2	14.2	7.5	11.1	6.8	3.4	1.1
Bacteria	2.3	1.1	10.5	6.6	15.2	7.4	11.8	6.9	3.6	1.0
Eukarya	2.1	0.9	13.2	8.1	17.5	8.7	14.4	8.4	3.8	1.0

Each sequence was shuffled 5 times

On the other end of the spectrum, we consider comparative structures for the large subunit (lsu) and small subunit (ssu) ribosomal rna. We see that ssu rrna exhibits long measures of end-to-end distance. The ssu rrna structures often contain a pseudoknot in the exterior loop. Pseudoknots may provide an evolutionary strategy for increasing end-to-end distances (Ermolenko and Mathews 2020), meaning in this case, the biology actually pushes the ends further apart. From Table 7, we see a degree of exactly 4 for every ssu rrna sequence we study, and UNP values that are higher than for any other family.

The behavior for lsu rrna is more nuanced. Collectively, the family has a high variance among most parameters. However, when the dataset is broken into the lsu rrna from archaea, bacteria, and eukarya, a different story emerges: all 13 of the bacteria sequences have small end-to-end distance parameters, supported by degree one external loops. The 7 archaea sequences almost all have small parameters, but a single outlier drives up the means and the variances. Finally, the eukarya are split: 13 out of 18 sequences have very high values of DEG and UNP, and the remaining 5 have low values. This bimodality, clearly visible in the graphs in Sect. C. 2, contributes to unusually high variances.

The low end-to-end distance measures for archaea and bacteria, as well as the high end-to-end distance measures for eukarya, are additionally supported by the consensus structures in Rfam (Griffiths-Jones 2003). Even for the eukarya sequences, the ETE values are on average less than 8nm.

The sequences we studied come from curated collections of ssu rrna sequences from Konings and Gutell (1995) and lsu rrna sequences from Fields and Gutell (1996). From the sequences in these papers, we took all sequences that had complete structures available in the Comparative RNA Web-2 website (Cannone et al. 2002; Chan et al. 2023), which left us with 54 ssu rrna sequences and 38 lsu rrna sequences. Although the ArchiveII dataset does include some ssu and lsu ribosomal rna structural information, each structure is split into multiple domains.

The ssu rrna structures frequently contain pseudoknots in the exterior loop. There are several computational methods for pseudoknot removal, with no one being convention (Smit et al. 2008). The impact of choosing different pseudoknot removal methods on our characteristics of end-proximity is unclear. So, to assess DEG and UNP in this context, we computed the shortest path in each structure from the $5^{\prime }$ end to the $3^{\prime }$ end, and then counted unpaired bases and base pairs in this path. We omit computing stem length STM for these pseudoknotted structures.

Upon randomly shuffling each sequence five times, the distributions of each measure again moved closer to the theoretical Pfold distributions. The statistics for each shuffle look strikingly similar to those of the mrna and lncrna in Table 4.

## Exterior Helices and Stems

**Table 8:** Parameters describing the first helix or stem in a secondary structure

Parameter	Definition
HEL	Number of base pairs in the first helix of a secondary structure
STM	Number of base pairs in the first stem of a secondary structure
HSTM	Number of helices in the first stem

The analysis in Sect. 3 repeatedly revealed small means across all characteristics of end-proximity and all models of rna secondary structures. In Sects. 5 and 6 above, we also saw that real-world rna datasets consistently display low measures of end-proximity. Hence, across the entire spectrum from Fig. 1, we should expect the ends of rna sequences to be close. This leads to the question: is there a different biological feature that would better distinguish real-world rna from our simplified combinatorial branching models?

One possibility is the number of base pairs in helices in the exterior loop of an rna secondary structure. We call such helices *exterior helices*. We are particularly motivated by the fact that some families like 5 S rrna and trna can be very well-structured with almost all representatives having degree one exterior loops with almost no variability. These factors led us to consider the length of exterior helices as another biological feature that could help make ends meet (Table 8).

In general, we focus on the first helix in a secondary structure when reading from the $5^{\prime }$ end to the $3^{\prime }$ end of the strand. Let HEL represent the number of base pairs in the helix with a nucleotide closest to the $5^{\prime }$ end of the strand.

**Fig. 7:**
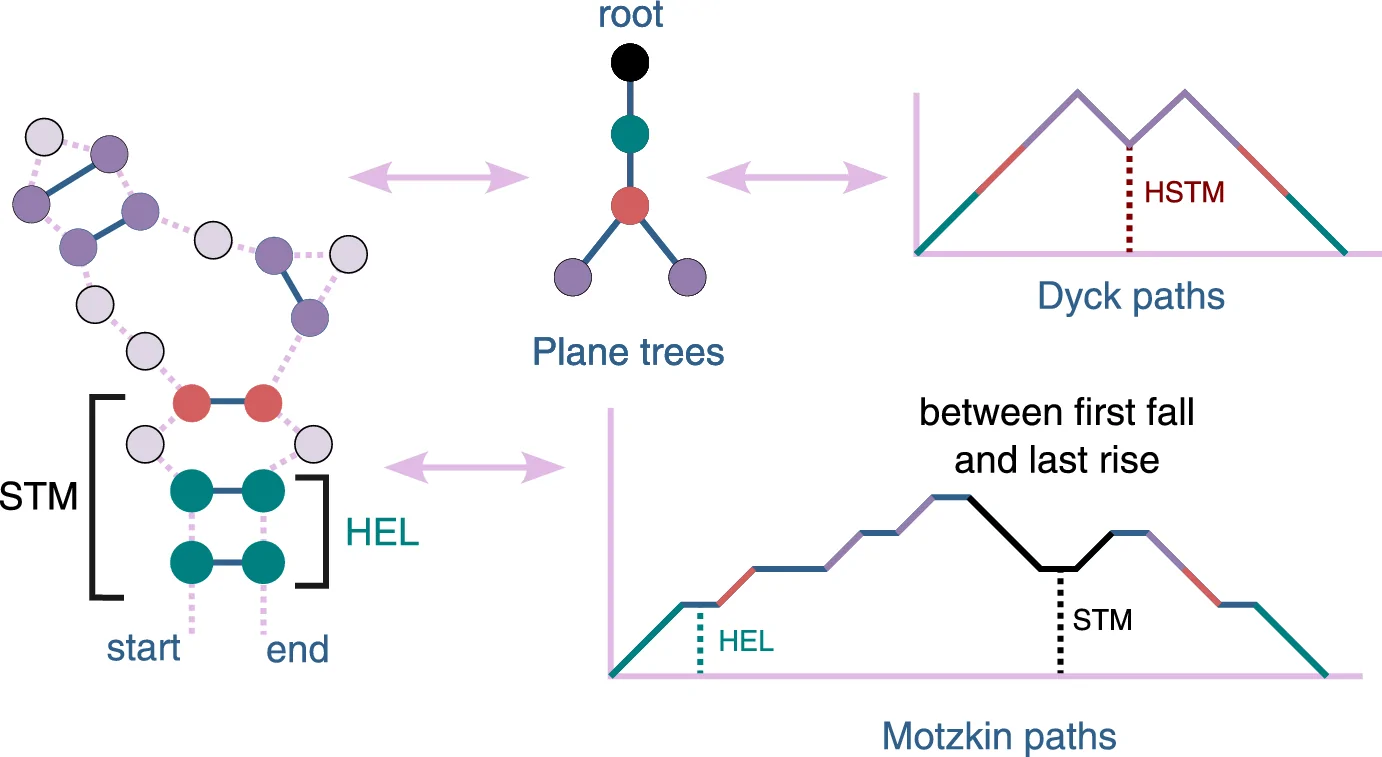
The length of the first helix HEL in an rna secondary structure is equal to the depth of the first mountain in the corresponding Motzkin path. Here, depth is defined as the height of the lowest valley ($\searrow \nearrow$) or plateau (→) in the path. When restricting to the region of the mountain between the first fall and last rise, the height of the lowest valley or plateau gives the length of the stem, STM. In the Dyck path representation, the depth corresponds to the number of helices in the stem, HSTM(Color figure online)

Sometimes, the first helix may be interrupted by bulges or interior loops but it may be part of a longer chain of helices that still adds stability to the ends of the secondary structure. In Sect. 6 above, this was particularly true for the family of SRP rna in the ArchiveII dataset (see Table 6). Thus, we consider the number of base pairs in the first stem of a secondary structure, denoted STM. Two base pairs (*i*, *j*) and (*k*, *l*) with i<k belong to the same stem if i<k<l<j and there is no base pair (*a*, *b*) with i<a<b<k or l<a<b<j. On the level of plane trees, the first stem in a secondary structure includes the first helix (represented by a single node) and all descendants of that node up to and including the first descendant that does not have exactly one child. See Fig. 7 for an example.

Finally, we also consider the number of helices comprising the first stem, called HSTM. We will find a type of asymptotic independence in our Motzkin path models below: for the limiting means of each distribution, $\mathbb {E}(\texttt {HSTM}) \times \mathbb {E}(\texttt {HEL}) = \mathbb {E}(\texttt {STM})$. Intuitively, in the limit, the average number of base pairs in the stem is equal to the average number of base pairs in each helix times the average number of helices in the stem. This suggests that asymptotically, the length distribution of the closing helix is no different than for any other helix in the first stem.

We note that the plane tree representation of secondary structures compresses helices into single nodes, so it is only possible to investigate HSTM in this model. However, when representing plane trees or Motzkin paths as dot-bracket sequences, we have a duality: because single pairs of parentheses represent entire helices in the plane tree representation, the number of parentheses in the first maximal set of consecutive paired parentheses represents HSTM in the plane tree model, and HEL in the Motzkin model. More on this connection appears in Appendix A.

In Sect. 7.1 below, we investigate the distribution of HEL, STM, and HSTM under the branching models defined by Dyck paths and Motzkin paths. Then, in Sect. 7.2, we examine the distribution of HEL under the Pfold grammar.

We find a substantial break in the models here: the expected limiting value of HEL is 9/8 for the Motzkin path model, but it is 4.7 for the Pfold grammar. In other words, the model that incorporates biological characteristics of branching structures also predicts much longer first helices than a typical branching structure. Finally, we compare to the datasets of rna from Sect. 5 and Sect. 6 in Sect. 8.

### Exterior Helices and Stems in Dyck and Motzkin paths

Before stating our results for HEL, STM, and HSTM for the Dyck and Motzkin path models, we first reframe each parameter explicitly in terms of paths, as illustrated in Fig. 7. Recall from Sect. 1.1 that a mountain in a Dyck or Motzkin path is any minimal span of steps where the path begins and ends on the horizon, leaving the horizon in between.

To identify HEL from a Motzkin path, restrict attention to the first left-to-right mountain and look for the lowest horizontal step (a “plateau”) or the lowest consecutive occurrence of $\searrow \nearrow$ (a “valley”). When a plateau or valley exists, the height of the lowest among them is HEL, where the height is defined to be the vertical distance to the horizon. If no such plateau or valley exists, this means the mountain goes straight up and then straight down, and the height of the mountain peak is HEL. Similarly, in a Dyck path, the height of the lowest valley is HSTM, or if no such valley exists, the height of the mountain peak is HSTM. Whether Dyck or Motzkin paths, we refer to the height corresponding to HEL or HSTM as the *depth* of the mountain.

To identify STM from a Motzkin path, again focus on the first mountain. Ignoring horizontal steps, if the mountain only travels upwards then downwards, then the height of the top of the mountain is STM. Otherwise, restrict attention to the portion of the mountain that is between the first ↘ step and the last ↗ step. The height of the lowest plateau or valley in this interval corresponds to STM.

In the results below, we rely on Proposition 2 again to discover the asymptotic distribution of HEL, STM, and HSTM. In all cases, the resulting distributions are now geometric, or equivalently, a negative binomial with only one coin flip.

#### Proposition 9

Consider the uniform distribution over all Dyck or Motzkin paths of length *n* as $n \rightarrow \infty$.

1. For Dyck paths, HSTM tends to $1 + {{\,\textrm{NB}\,}}(1, 3/4)$ with mean 4/3 and variance 4/9.
2. For Motzkin paths, HEL tends to $1 + {{\,\textrm{NB}\,}}(1, 8/9)$ with mean 9/8 and variance 9/64.
3. For Motzkin paths, HSTM tends to $1 + {{\,\textrm{NB}\,}}(1, 27/32)$ with mean 32/27 and variance 160/729.
4. For Motzkin paths, STM tends to $1 + {{\,\textrm{NB}\,}}(1, 3/4)$ with mean 4/3 and variance 4/9.

#### Proof

Our goal is to find a recurrence relation for each GF that tracks the corresponding depth of the paths.

Let *D*(*z*, *y*) be the GF which tracks Dyck paths by length (*z*) and height (*y*), where length is again the number of pairs of parentheses. In order to find the recurrence for *D*, we will need to define an auxiliary GF *H*(*z*, *y*), described momentarily. We begin with the standard Dyck path recurrence relation, modified slightly to include this helper GF:

D(z,y)=1+[zyH(z,y)]D(z,1)

**Fig. 8:**
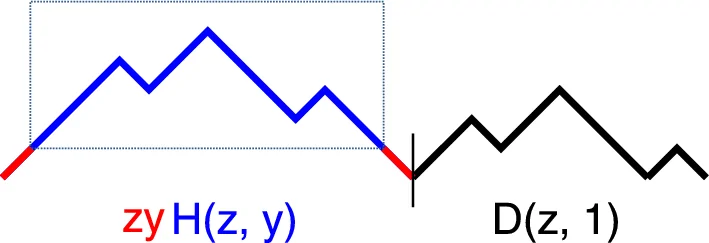
A decomposition of Dyck paths according to the first return to the line y=0. The Dyck path within the box adds to the length of the first helix only if its first and last position are paired (Color figure online)

Overall, this recurrence is motivated by decomposing a Dyck path according to its first return to the *x*-axis, as in Fig. 8. A Dyck path is either empty (contributing a 1), or it can be broken into three pieces: a pair of parentheses enclosing a Dyck path, *zy*; the Dyck path in between the parentheses, *H*(*z*, *y*); and another Dyck path, *D*(*z*, 1).

Now, we explain why the terms are *H*(*z*, *y*) and *D*(*z*, 1): for *D*(*z*, 1), this is the univariate generating function for all Dyck paths by length, which we include because the Dyck path at the end of the recurrence cannot contribute to the depth of the first mountain. Then, we define *H*(*z*, *y*) to count all Dyck paths by length (*z*) and depth (*y*), except Dyck paths with 2+ mountains (or with zero) are now counted by coefficients of the form $z^i y^0$. This is necessary because the depth of a path with 2+ mountains will be 1 after the path is enclosed by the set of parentheses in the recurrence, and should be counted in the overall generating function *D*(*z*, *y*) by the coefficient of $z^{i + 1} y^{0 + 1}$. In other words, *H*(*z*, *y*) only assigns positive depth to Dyck paths whose first step is paired with its last step. With this interpretation of *H*, we obtain the recurrence,

H(z,y)=(1-z)D(z,1)+zyH(z,y),

where the (1-z)D(z,1) term counts Dyck paths whose first step is not paired with its last step, and the *zyH*(*z*, *y*) term counts the rest.

Solving this system reveals that the GF *D*(*z*, *y*) is of the form required in Proposition 2 with g(y)=y, $a(z, y) = y(z^2 - 1)$, and ρ=1/4. Plugging into the proposition yields the limiting distribution encoded by 3y/(4-y), which is the PGF for a geometric random variable with parameter p=3/4.

Paralleling the changes for the Dyck path specification, let *M*(*z*, *y*) be the bivariate GF enumerating Motzkin paths by length (*z*, now counting individual nucleotides) and by depth of the first left-to-right mountain (*y*). Also, let *H*(*z*, *y*) be the equivalently defined helper function. Then, the following specification holds:

$$\begin{aligned} M(z, y)&= 1 + zM(z, y) + z^2yH(z, y)M(z, 1),\\ H(z,y)&= z^2yH(z, y) + (1-z^2)M(z, 1). \end{aligned}$$

Solving for *M*(*z*, *y*) again reveals a GF fitting the requirements of Proposition 2, this time with g(y)=y, $a(z, y) = z^2(yz^3 - yz^2 - z + 1)$, and ρ=1/3. The proposition gives the limiting GF (8y/9)/(1-y/9), which again corresponds to a geometric random variable, this time with parameter p=8/9.

For the length and number of components of the stem in a Motzkin path, we develop two grammars that output all dot-bracket sequences exactly once but allow us to track additions to the stem. First, for STM, we have the start symbol $M_f$ corresponding to when the grammar has yet to start generating the first stem. Then, $M_*$ is the symbol corresponding to the first stem being in the middle of production, and *M* is the symbol for when the grammar has moved past producing the stem. Additionally, we have a symbol *L* that corresponds to adding any number of consecutive dots. We obtain:

$$\begin{aligned} M_f&\rightarrow \emptyset \ \big |\ .M_f\ \big |\ (M_*)M\\ M_*&\rightarrow L\ \big |\ L(M_*)L\ \big |\ L(M)L(M)M\\ M&\rightarrow \emptyset \ \big |\ .M \ \big |\ (M)M\\ L&\rightarrow \emptyset \ \big |\ .L \end{aligned}$$

Here, ∅ represents outputting nothing. The symbols $M_f$ and *M* both have production rules modeled after the standard recursion for Motzkin paths. In contrast, $M_*$ has rules broken into whether the output has no parentheses, *L*; a single outermost pair of parentheses, $L(M_*)L$; or more than one outermost pair of parentheses to break the stem, *L*(*M*)*L*(*M*)*M*. Using the Chomsky-Schützenberger theorem once more, this converts to the system of equations

$$\begin{aligned} M_f&= 1 + zM_f + z^2uM_*M,\\ M_*&= L + z^2uL^2M_* + z^4L^2M^3\\ M&=1 + zM + z^2M^2\\ L&= 1+zL \end{aligned}$$

This is solved via Gröbner bases in our SageMath worksheet, leading to a generating function where Proposition 2 applies. The limiting GF is 3u/(4-u), again corresponding to the PGF for a geometric random variable with p=3/4.

The grammar needs a slight modification to track the number of helices in the stem. In the rules for $M_*$, we now need to know more information about the production $M_* \rightarrow L(M_*)L$: if both *L*s are empty, a pre-existing helix is growing longer, but if either *L* is non-empty, a bulge or interior loop causes a new helix to be formed in the stem instead. This change corresponds to replacing the equation for $M_*$ with

$$ M_* = L + z^2M_* + z^2u(2(L-1) + (L-1)^2)M_* + z^4\,L^2\,M^3, $$

where the $z^2M_*$ term corresponds to both *L*s being empty, the $z^2u2(L-1)M_*$ term represents exactly one of the left or right *L* being empty, and the $z^2u(L-1)^2M_*$ represents both *L*s being non-empty. Every other part of the analysis is the same, and the limiting PGF is now 27u/(32-5u), corresponding to the geometric random variable with parameter p=27/32. □

In the result above, we note that the distribution of HEL in Dyck paths is the same as the distribution of STM for Motzkin paths. See Appendix A for more on this connection. Additionally, we note that the results imply an asymptotic independence: the mean number of components in the first stem times the mean length of the first helix is $32/27 \cdot {9/8} = 4/3$, the mean length of the stem.

### Closing Helix Length in the Pfold Grammar

The statement for the Pfold grammar is again analogous to Dyck and Motzkin paths. The primary challenge is finding an equivalent form of the Pfold grammar that allows one to track the length of the first helix.

#### Proposition 10

Define ρ as the unique smallest positive solution to $R(z) = (1 - p_1q_2z)^2(1-p_3z^2) - 4p_2q_1q_2q_3z^3$. In the Pfold grammar, HEL has a limiting distribution of $1 + {{\,\textrm{NB}\,}}(1, 1-p_3\rho ^2)$. Under the default parameters, the mean is 4.7 and the variance is 17.5.

#### Proof

As in Proposition 7, we begin with the three production rules [1], [2],  and [3], and we add two new symbols $S_f$ and $F_f$ that track when the grammar is still outputting the first helix versus when it has moved past the helix. So, symbols without the tag ‘*f*’ correspond to productions that cannot impact the first helix. Then, we add the two rules:

$$ [1'']\ S_f \rightarrow (F_f)S \ (p_1p_2) \ \big | \ .S_f \ (p_1q_2) \ \big | \ (F_f) \ (q_1p_2) \ \big | \ . \ (q_1q_2) $$

$$ [2'']\ F_f \rightarrow (F_f) \ (p_3) \ \big | \ LS \ (q_3) $$

Rule $\left[1^{\prime \prime }\right]$ was derived by combining rules [1] and [2] in the original grammar, which is necessary in order to track whether the initial symbol *L* in the rule S→LS becomes the first helix or whether it becomes a dot, in which case the remaining *S* may still become the first helix.

From this point onwards, the analysis pipeline matches that of Proposition 7, and details are provided in the SageMath worksheet. □

## Making Ends Meet

**Table 9:** HEL, STM, and HSTM distribution statistics across natural and artificial rna datasets, with the theoretical Pfold and Motzkin distributions for comparison

Seq.	HEL		STM		HSTM		HEL		STM		HSTM	
	μ	σ	μ	σ	μ	σ	μ	σ	μ	σ	μ	σ
**m** RNA **/lnc** RNA	**Natural dists**						**Artificial dists**					
RPL41A	3.7	0.8	5.1	1.9	1.4	0.7	5.0	2.0	12.6	9.4	2.8	2.2
ATP	4.4	0.6	10.8	1.2	3.2	0.5	4.4	1.8	10.3	7.4	2.5	1.8
HSBP1	6.4	1.3	6.9	1.7	1.2	0.6	4.9	2.0	12.3	8.7	2.7	2.1
MIF	6.6	1.1	7.3	1.3	1.2	0.4	4.6	2.0	11.9	8.5	2.8	2.1
Bglobin	3.7	0.6	8.4	1.7	2.0	0.4	4.9	1.9	12.5	9.3	2.8	2.2
MRP51	3.9	0.9	13.2	1.2	2.2	0.4	4.8	2.1	12.4	9.1	2.7	2.1
GAPDH	3.1	1.7	5.8	1.8	1.8	0.6	4.7	1.9	11.6	8.9	2.7	2.1
FLucif	4.0	1.8	5.8	3.1	1.4	0.6	5.0	2.1	11.7	8.5	2.6	1.9
Hotair	2.8	0.9	19.9	4.0	5.0	1.0	4.6	2.0	10.7	8.1	2.5	2.0
Neat1	6.3	1.4	9.4	3.0	2.3	1.3	5.1	2.3	13.4	10.1	2.8	2.2
Combined	4.5	1.8	9.3	4.9	2.2	1.3	4.8	2.0	11.9	8.9	2.7	2.1
**ArchiveII**	**Natural dists**						**Artificial dists**					
trna	6.8	0.8	6.9	0.6	1.1	0.2	4.3	1.8	12.2	7.0	3.1	1.9
SRP rna	5.7	3.5	17.7	13.9	4.1	3.0	4.4	1.9	12.7	9.1	3.1	2.3
5 S rrna	8.2	2.3	9.1	1.3	1.2	0.4	4.3	1.8	12.5	8.9	3.2	2.4
RNaseP	5.4	3.7	8.5	2.9	1.8	0.6	4.5	1.9	11.1	8.7	2.7	2.2
tmrna	7.0	0.3	7.2	2.2	1.0	0.4	4.6	2.0	11.8	9.2	2.7	2.2
Combined	6.8	2.8	10.6	8.3	1.9	2.0	4.4	1.9	12.2	8.7	3.0	2.2
SSU/LSU **r**RNA	**Natural dists**						**Artificial dists**					
ssu	5.0	0.0	–	–	–	–	4.8	2.1	12.8	8.8	2.9	2.0
lsu (Combined)	7.3	3.0	–	–	–	–	5.1	2.3	12.5	9.6	2.8	2.3
Archaea	5.6	2.6	–	–	–	–	4.1	1.4	13.1	12.1	3.3	3.2
Bacteria	7.9	0.3	–	–	–	–	4.8	1.9	12.4	8.2	2.9	2.1
Eukarya	7.6	3.9	–	–	–	–	5.6	2.6	12.4	9.5	2.6	1.9
Combined	6.0	2.2	–	–	–	–	4.9	2.2	12.7	9.1	2.9	2.1
*Theoretical distributions*												
Pfold	4.7	4.2	–	–	–	–	–	–	–	–	–	–
Motzkin	1.1	0.4	1.3	0.7	1.2	0.5	–	–	–	–	–	–

Because HEL and STM are proxies for the thermodynamic stability of the exterior loop, they give an indication of whether the structures are simply meeting at the ends, or whether they are made to meet. We have already seen in Sect. 7.1 that a branching structure under the Motzkin model has a HEL mean of only 1.1, suggesting these structures just meet at the ends. In contrast, the Pfold grammar reweights branching structures to be more biologically realistic and has a HEL mean of 4.7, thereby implying the ends are made to meet.

The mrna and lncrna natural distributions have mean HEL values that are in line with the Pfold grammar as well their artificial counterparts, as seen in Table 9. This shows that the helices are longer than expected for a random branched structure, and these ends are made to meet. However, it also implies that the Pfold model of HEL cannot distinguish any additional biological constraints that impact the behavior of the ends of these sequences.

In comparison to the theoretical distributions, the ArchiveII dataset families have ends that are “bound to meet.” Combined with the short ETE values from Sect. 6, each of these families of rna has a mean value of HEL that surpasses the theoretical Pfold value. Although SRP and RNaseP have relatively short first helices, their stems are among the longest in the set. For SRP, the consensus structures in Rfam (Griffiths-Jones 2003) display very little branching, explaining the high values of STM.

Finally, the ssu rrna sequences have consistent first helices that are in line with the Pfold default, suggesting that this helix length is not responsible for the unusually large ETE distances for this dataset. For the lsu rrna, the bacteria and eukarya sequences were high and low ETE outliers, respectively. Here, we see that these sequences also have particularly long first helices, whereas the archaea sequences have both more typical HEL and ETE values. This suggests that when the ETE values are particularly short or particularly long, they may be reinforced via long helix lengths.

When comparing the natural and artificial distributions for HEL and STM, the behavior is similar to the characteristics of end proximity studied in Sects. 5 and 6. The natural distributions tend to be in concentrated wells, where as the artificial distributions tend to have consistent means and variances. See side-by-side comparisons in Sects. C. 3 and C. 4.

## Conclusion

The multivariate analytic combinatorics pipeline used here has enabled us to fully characterize the distributions of several parameters of rna secondary structures. While the means of other characteristics of rna have been investigated previously (Hofacker et al. 1998), the pipeline here brings richer information about end-to-end distance distributions. In particular, the ETE parameter was based off of an experimentally validated model that converts the degree and number of unpaired bases in an rna exterior loop into an approximate end-to-end distance measurement in nanometers (Aalberts and Nandagopal 2010; Lai et al. 2018). But, analyzing the distribution of ETE requires analyzing the distributions of DEG and UNP simultaneously, since these parameters are not independent. By using three parameters, our trivariate generating functions enabled tracking the distribution of ETE in the combinatorial models to arbitrary precision.

By comparing the Pfold, Dyck, and Motzkin distributions for ETE, we find that DEG plays the primary role in determining ETE, while UNP plays a much smaller role. For example, although the mean number of unpaired nucleotides UNP differed by more than 10 in the Motzkin distribution versus the Pfold distribution, the mean value for ETE differed by less than 1 nanometer. Additionally, in our data analysis from Sect. 5, the family with the largest ETE measurement is the ssu rrna  where pseudoknots are known to interrupt the branching of the exterior loop.

As seen in Sect. 8, some rna structures have shorter first helices but longer first stems. Refining the combinatorial specifications for the Pfold grammar could yield more information on stems and whether their length accounts for the large number of rna sequences with degree one exterior loops. Initial testing shows that the additional symbols needed to track stem length greatly increase the computational complexity in the corresponding system of equations defining the GF.

As is often the case with research at the interface of mathematics and biology, the results here tie back to other well-studied combinatorial objects. In particular, Dyck paths can be encoded as pattern-avoiding permutations (Krattenthaler 2001; Adin et al. 2018), and the results here give distributional information on the blocks contained within a pattern. The results on the degree of the Motzkin paths connects to the number of blocks in a *Motzkin permutation* (Elizalde and Mansour 2005). Hence, the results here may have additional implications on properties of pattern-avoiding permutations.

Finally, the recurrence of the negative binomial distribution brings an obvious question: what does a coin flip represent combinatorially? If each tails represents increasing a measure of end-proximity by one, then is there a corresponding interpretation for a heads?

## Data Availability

The ArchiveII dataset (Sloma and Mathews 2016; Mathews 2019) and the ssu rrna and lsu rrna sequences (Cannone et al. 2002; Chan et al. 2023) are available online. The lncrna and mrna sequences from Lai et al. (2018) were obtained via personal communication with the authors. Verifications of our theoretical computations are available in a companion SageMath worksheet: https://github.com/gtDMMB/EndProximity

## Comparison of Dyck and Motzkin Path Limiting Distributions

From Proposition 1, we see that the limiting distribution of DEG is the same for Dyck and Motzkin paths. Similarly, in Proposition 9, the distribution of HSTM in Dyck paths is the same as the distribution of STM in Motzkin paths. Here, we outline a combinatorial argument for their equivalence.

Motzkin paths can be compressed to Dyck paths by removing all horizontal steps from a Motzkin path. (As described below, this does not lead to Dyck paths with the usual rna interpretation.) Under this compression, infinitely many Motzkin paths map to the same Dyck path. However, if we restrict our attention to Motzkin paths of length *n* with n-k horizontal steps, these map to Dyck paths of length *k*. Moreover, every Dyck path of length *k* is hit by exactly the same number of Motzkin paths: to see this, note that any such Dyck path with *k* steps can be expanded back to a Motzkin path with *n* steps by arranging the horizontal steps among the non-horizontal steps in $$\left( {\begin{array}{c}n\\ k\end{array}}\right) $$ ways.

Under this compression, DEG remains the same in both the original Motzkin path and the corresponding Dyck path. For this reason, we expect the limiting distribution of DEG to be the same for both. Similarly, STM in a Motzkin path corresponds to the first maximal set of consecutive paired parentheses in the Dyck path, which describes HSTM.

It may be tempting to compare limiting distributions by using the compression from Motzkin paths to Dyck paths that corresponds to the two representations of rna. On the level of dot-bracket sequences, this means first shrinking all consecutive runs of paired parentheses into a single pair of parentheses, and then removing all dots. However, this compression strategy fails to preserve limiting distributions because each Dyck path no longer corresponds to the same number of Motzkin paths. The issue arises when thinking of the reverse process: when a Dyck path is being expanded into a Motzkin path, dots can only be placed in locations that split helices as necessary. For instance, ‘(())’ cannot be expanded into ‘.(())’, because the latter compresses to just ‘()’.

For example, consider compressing dot-bracket sequences of length five with two helices down to parenthesizations with two helices. We have the following correspondence:

$$\begin{aligned}&\{.()(), \quad \qquad (.)(), \quad \qquad ().(), \quad \qquad ()(.), \quad \qquad ()().\} \leftrightarrow ()()\\&\quad \{(.()), \quad \qquad (().)\} \leftrightarrow (()) \end{aligned}$$

Here, the number of dot-bracket sequences corresponding to ()() is over double that for (()).

Ultimately, this means that the Motzkin paths are not spread uniformly over the Dyck paths by this compression strategy, and so the limiting distributions are not necessarily preserved through this compression. This explains why the limiting distribution of HSTM in Motzkin paths does not correspond to HSTM in Dyck paths.

## ETE for Motzkin Paths

Here, we complete the proof of Corollary 6 by computing the mean and variance of ETE.

### Proof

Given the limiting bivariate PGF $P(u, v) = v/(3-u-v)^2$ from Lemma 5, we first find an explicit formula for the probability mass function for the joint distribution. To do so, we expand *P*(*u*, *v*) as a series using that $1/(1-x)^2 = \sum _{n = 0}^\infty (n + 1)x^n$:

$$\begin{aligned} \frac{v}{(3-u-v)^2}&= \frac{v}{9} \left( \frac{1}{1 - \frac{u + v}{3}}\right) ^2\\&= \frac{v}{9} \sum _{n = 0}^\infty (n + 1) \left( \frac{u + v}{3}\right) ^n\\&= \sum _{n = 0}^\infty \sum _{k = 0}^n \left( {\begin{array}{c}n\\ k\end{array}}\right) \frac{n + 1}{3^{n + 2}} u^k v^{n - k + 1}\\&= \sum _{i = 0}^\infty \sum _{j = 0}^\infty \left( {\begin{array}{c}i + j - 1\\ i\end{array}}\right) \frac{i + j}{3^{i + j + 1}} u^i v^j. \end{aligned}$$

Collectively, this tells us:

$$ p_{i, j}:= \mathbb {P}(\texttt {UNP}= i, \texttt {DEG}= j) = \left( {\begin{array}{c}i + j - 1\\ i\end{array}}\right) \frac{i + j}{3^{i + j + 1}}. $$

With the approximate distance formula $\texttt {ETE}= \sqrt{1.5^2 \cdot \texttt {DEG}^{6/5} + 0.62^2 \cdot \texttt {CHN}^{6/5}}$, we can now numerically compute $\mathbb {E} (\texttt {ETE})$ and $\mathbb {E} (\texttt {ETE})^2$. We have:

B1 $$\begin{aligned} \mathbb {E}(\texttt {ETE}) = \sum _{i=0}^\infty \sum _{j=0}^\infty \sqrt{1.5^2 \cdot j^{6/5} + 0.62^2 \cdot (i+j-1)^{6/5}} \cdot p_{i, j}. \end{aligned}$$

We claim that summing the terms through i+j=50 yields the expected value to within 0.0025. To see this, we give a rough bound the tail of this sum:

$$ T_k:= \sum _{i + j \ge k} p_{i, j}\sqrt{1.5^2 \cdot j^{6/5} + 0.62^2 \cdot (i+j-1)^{6/5}}. $$

Our goal is to compare $T_k$ to the tail of series that are derivatives of geometric series, because these tails can be computed explicitly.

First, note that $$\left( {\begin{array}{c}i + j - 1\\ i\end{array}}\right) \le 2^{i + j - 1}$$ for all *i* and *j*. Thus, $p_{i, j} \le (2/3)^{i + j + 1}(i + j).$ Additionally, from the triangle inequality,

$$ \texttt {ETE}\le 1.5 (\texttt {DEG})^{3/5} + 0.62(\texttt {CHN})^{3/5} \le 2.12(\texttt {DEG}+ \texttt {UNP})^{3/5}. $$

Combining these two inequalities shows that

$$\begin{aligned} T_k&\le \sum _{i + j \ge k} (2.12)(i + j)^{8/5} \left( \frac{2}{3}\right) ^{i + j + 1} = 2.12\sum _{n = k}^\infty (n + 1)n^{8/5}\left( \frac{2}{3}\right) ^{n+1} \\&\le 2.12 \cdot 2/3 \cdot \sum _{n = k}^\infty n^3 \left( \frac{2}{3}\right) ^{n}, \end{aligned}$$

where the last inequality is true for k≥3 because $n + 1 \le n^{7/5}$ whenever n≥3. Temporarily replace 2/3=x inside the sum. Then, using common geometric series formulas,

$$\begin{aligned} 2.12 \cdot 2/3 \cdot \sum _{n = k}^\infty n^3 x^{n}&= 2.12 \cdot 2/3 \cdot \sum _{n = 0}^\infty (n + k)^3 x^{n + k}\\&= 2.12 \cdot 2/3 \cdot x^k \cdot \left[ \sum _{n = 0}^\infty n^3 x^n + 3k \sum _{n = 0}^\infty n^2 x^n + 3k^2 \sum _{n = 0}^\infty nx^n \right. \\&\quad + \left. k^3 \sum _{n = 0}^\infty x^n \right] \\&= 2.12 \cdot 2/3 \cdot x^k \cdot \left[ \frac{k^3}{1 - x} + \frac{3k^2x}{(1-x)^2} + \frac{3kx(1 + x)}{(1-x)^3} \right. \\&\quad \left. + \frac{x(1 + 4x + x^2)}{(1-x)^4}\right] . \end{aligned}$$

Replacing x=2/3 once more yields

$$\begin{aligned} T_k&\le 2.12 \cdot 2/3 \cdot \sum _{n = k}^\infty n^3 x^{n}\\&\le 2.12 \cdot 2 \cdot \left( \frac{2}{3}\right) ^k \left( k^3 + 6k^2 + 30k + 74\right) \\&\le 2.12 \cdot 2 \cdot \left( \frac{2}{3}\right) ^k \cdot 2k^3, \end{aligned}$$

where the last equality is true for k≥10. It is easy to verify that this bound is also decreasing for k≥10, and for k=50, is less than 0.0025. Thus, it suffices to sum Eq. (B1) over all i,j≤50 to achieve the mean to within 0.0025.

For the second moment,

$$ \mathbb {E}(\texttt {ETE})^2 = \sum _{i=0}^\infty \sum _{j=0}^\infty \left( 1.5^2 \cdot j^{6/5} + 0.62^2 \cdot (i+j-1)^{6/5}\right) \cdot p_{i, j}, $$

and a similar argument shows that summing among terms where i+j≤60 gives an error less than 0.001. Hence, we can calculate the expressed variance using the shortcut formula $\mathbb {E}(\texttt {ETE})^2 - [\mathbb {E}(\texttt {ETE})]^2$ with an appropriate increase in precision for the estimate of $\mathbb {E}(\texttt {ETE})$ to ensure the error of $(\mathbb {E}(\texttt {ETE}))^2$ remains small. □

## Graphs of Distributions of Parameters in Datasets

### STM distributions

We did not assess the first stem length in the 16 S and 23 S sequences due to pseudoknots in the exterior loop.

## Footnotes

1 Two base pairs (*i*, *j*) and (*k*, *l*) with i<k form a pseudoknot if i<k<j<l. Although such crossed pairings are known to exist, and to be functionally important, their prediction remains a challenging aspect of rna pairwise interactions. Hence, they are typically considered part of the tertiary interactions that, along with base triples, coaxial stacking, etc., ultimately determine the three dimensional molecular conformation.

2 Although non-canonical pairings are known to exist, they are treated as a small internal loop under the most common secondary structure prediction methods which are based on the nearest neighbor thermodynamic model (NNTM) (Turner and Mathews 2009).

3 The Pfold grammar probability parameters were estimated from a dataset of established tRNA and large subunit ribosomal rna (Knudsen and Hein 2003).

4 David Mathews, personal communication.

5 Interpretations of the monomer separation “links” in the approximation formula (Aalberts and Nandagopal 2010) vary. While we have used $\texttt {ETE}= \sqrt{b^2 \texttt {DEG}^{6/5} + c^2 \texttt {CHN}^{6/5}}$, as illustrated in Fig. 2, the authors of Lai et al. (2018) used $\texttt {ETE}= \sqrt{b^2 \texttt {DEG}^{6/5} + c^2 \texttt {UNP}^{6/5}}$. Since $\texttt {CHN}= \texttt {DEG}+ \texttt {UNP}- 1$, this makes our estimates slightly larger whenever $\texttt {DEG}> 1$.
